# Iron acquisition strategies of the rock-inhabiting fungus *Knufia petricola* reveal vulnerability to chelator-based mitigation

**DOI:** 10.1128/aem.02311-25

**Published:** 2026-05-13

**Authors:** Ruben Gerrits, Julia Schumacher, Anna A. Gorbushina

**Affiliations:** 1 Bundesanstalt für Materialforschung und -prüfung (BAM) 42220, Berlin, Germany; 2 Freie Universität Berlin 9166, Berlin, Germany; Royal Botanic Gardens Kew, Surrey, United Kingdom

**Keywords:** siderophore, olivine, reductive iron assimilation, melanin, minimum inhibitory concentration, fungal iron uptake

## Abstract

Fungal iron acquisition has mostly been studied for pathogens living in competitive environments where securing iron from the host is critical for proliferation. However, how fungi from stress-rich but competition-free material surfaces handle iron uptake is unknown. We studied these processes in rock-inhabiting fungi, known for their constitutive melanin production, extremotolerant lifestyle, and colonization of exposed surfaces such as solar panels and marble monuments, by choosing *Knufia petricola* to represent this group. The characterization of targeted mutants showed that *K. petricola* can take up iron via an extracellular—yet undefined—siderophore and a ferroxidase-iron permease complex. By disrupting melanin synthesis, it was demonstrated that this pigment can reduce and adsorb iron, but nevertheless does not contribute significantly to iron acquisition. Moreover, chemotropism toward an iron source was found to be siderophore-dependent. Ferric citrate could not be directly assimilated. Finally, it was found that *K. petricola* and other rock-inhabiting fungi exhibited high sensitivity to strong iron chelators, such as EDTA. These observations not only offer a potential mitigation strategy for preventing fungal colonization of subaerial materials using iron chelators but also highlight the specialized adaptations of these fungi to low-competition environments.

**IMPORTANCE:**

The rock-inhabiting fungus *Knufia petricola* acquires iron primarily via reductive iron assimilation, independently of the iron-adsorbing and iron-reducing melanin. Its siderophore, although extracellular and allowing chemotropism toward an iron source, cannot obtain iron from strong iron chelators, such as BPS and EDTA. This sensitivity to iron sequestration opens avenues for mitigating the fungal colonization of substrates such as solar panels.

## INTRODUCTION

Iron, the fourth most abundant element in Earth’s upper continental crust (1), has, since the rise of atmospheric oxygen levels around 2.4 Gya (the Great Oxidation Event, GOA), started to oxidize and precipitate in the form of oxides (2–5). Imlay (6) noted that the abundant amounts of available iron pre-GOA resulted in the use of iron-sulfur clusters within many proteins, such as redox enzymes involved in respiration. Post-GOA, oxygen-enduring organisms started to thrive in aerobic environments by adopting strategies to cope with low iron availability and oxidative stress (6, 7).

Fungi may use up to four mechanisms to acquire iron from their environment (8–10): (i) uptake of ferric iron after its extracellular reduction by ferric reductases (FREs) and re-oxidation via a high-affinity ferroxidase-iron permease complex (reductive iron assimilation, RIA), (ii) uptake of ferric iron complexed by low-molecular-mass chelators called siderophores via high-affinity siderophore transporters (siderophore-mediated iron acquisition, SIA), (iii) uptake of heme-complexed iron, and (iv) uptake of ferrous iron by low-affinity transporters. Iron uptake is well studied in the budding yeast *Saccharomyces cerevisiae* and the opportunistic animal pathogen *Aspergillus fumigatus. S. cerevisiae* possesses one RIA complex localized in the plasma membrane (FTR1-FET3) (11) and one low-affinity ferrous iron transporter (FET4) (12), and although it does not synthesize siderophores, it has four siderophore iron transporters (SITs) for uptake of foreign siderophores (xenosiderophores) (13, 14). The filamentous fungus *A. fumigatus* contains one RIA complex (FtrA-FetC) (15), a low-affinity iron transporter (FetD) (10), and five putative SITs for taking up iron bound to (xeno)siderophores (16). Four hydroxamate siderophores are produced by non-ribosomal peptide synthetases (NRPSs): ferricrocin, hydroxyferricrocin, fusarinine C, and triacetylfusarinine C (17). Ferricrocin was initially assumed to function solely in intracellular iron handling (10, 17), but later studies showed its secretion and role in iron acquisition (18).

The mechanisms of iron acquisition, essential for growth and proliferation, have been primarily studied in pathogenic fungi to develop antifungal strategies in a medicinal or agricultural context (8, 10, 19–21). The active chelation of iron by the animal host hampers microbial infections (so-called nutritional immunity [22]), rendering the habitat of pathogenic fungi more iron-depleted than that of other fungi. We hypothesize, therefore, that the absence of competition for iron in their environmental niches renders saprobic fungi more sensitive to strong iron chelators, thereby offering a basis for potential mitigation strategies. We focused on the polyphyletic group of black fungi, which share similar morpho-physiological adaptations allowing them to survive in harsh environments, including melanized cell walls, slow yeast-like or meristematic growth, and the absence of complex reproduction cycles (23). While several black fungi are (opportunistic) animal pathogens (24), the group of rock-inhabiting fungi colonizes sub-aerial environments such as bare rocks in cold and hot deserts and human-made structures such as marble monuments and solar panels (25–27). The increasing use of the latter surfaces for energy generation (28), together with the considerable challenge in removing black fungal colonies or mitigating their growth (29), underscores the need for novel mitigation strategies.

Despite the high diversity, only a few genomes of rock-inhabiting fungi within the Arthoniomycetes, Eurotiomycetes, and Dothideomycetes have been sequenced to date. For even fewer fungi, genetic engineering tools for functional analyses are available. To address this gap, we selected *Knufia petricola* (Eurotiomycetes, Chaetothyriales, Trichomeriaceae) as a model organism for related rock-inhabiting fungi and developed tools for comparative phenotyping and genetic approaches (30–32). *K. petricola* constitutively produces the black 1,8-dihydroxynaphthalene (DHN) melanin and reddish carotenoids, which become visible in melanin-deficient mutants (30). Whether melanin, a multifunctional pigment, plays a role in iron acquisition, however, remains unknown.

Here, we combined bioinformatics and genetic analyses to reveal the basis of iron acquisition in *K. petricola*. Genes for two high-affinity iron uptake systems and DHN melanin synthesis were deleted to study the capacity of the systems to acquire iron from media or minerals. Accordingly, *K. petricola* primarily employs RIA over SIA, produces an (extracellular) siderophore which enables growth toward an iron source, and relies minimally on melanin, which plays no direct role in iron acquisition. The observation that *K. petricola* and other rock-inhabiting black fungi fail to grow in the presence of strong iron chelators demonstrates that iron chelation can serve as an effective and novel strategy to mitigate fungal colonization of exposed surfaces.

## RESULTS

### Genome analysis reveals genetic potential for RIA and SIA in *K. petricola*

**Fig 1:**
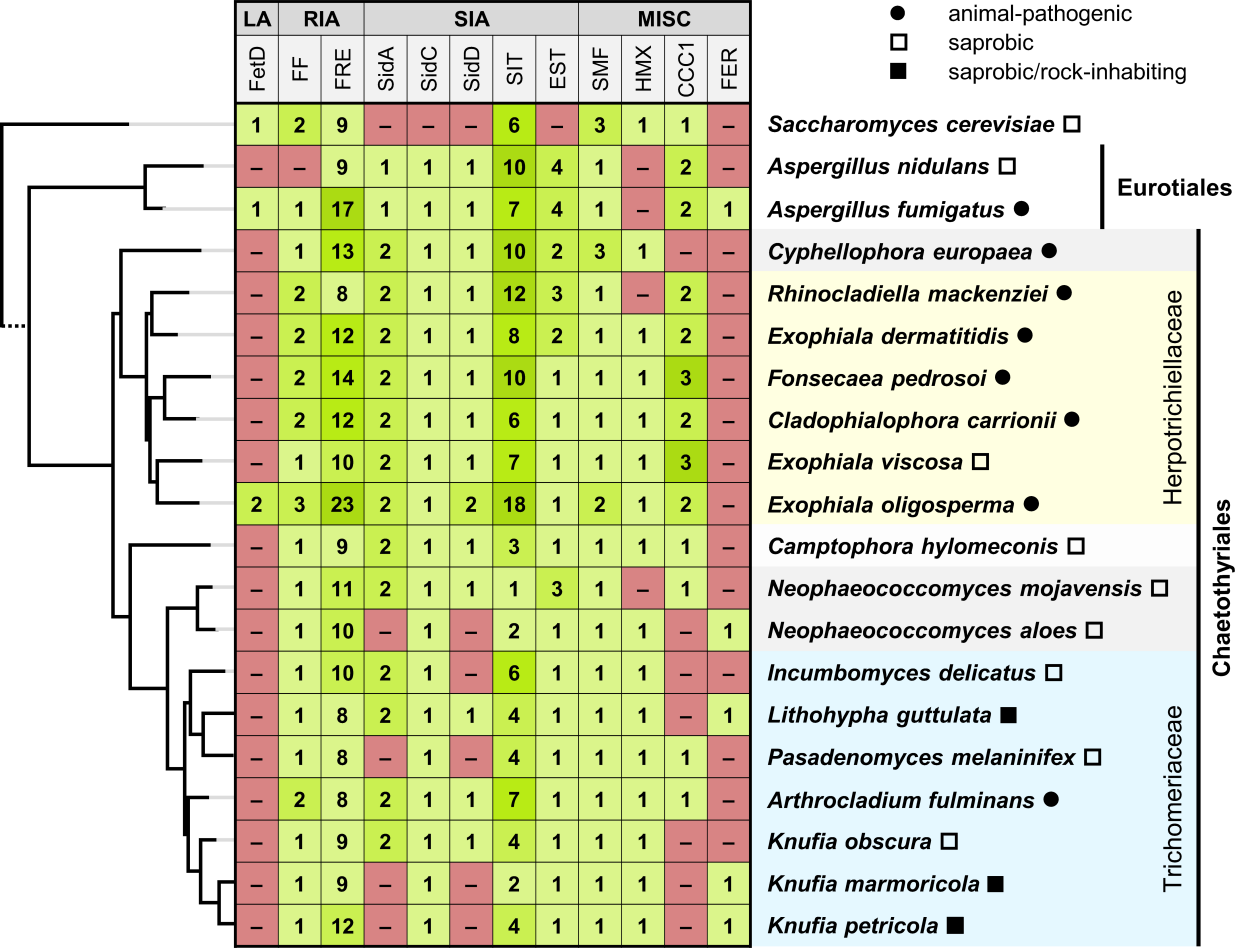
Distribution of iron acquisition genes in black fungi of the order Chaetothyriales. Representative Chaetothyriales species were selected based on phylogenetic diversity and isolation source. Species were categorized as pathogenic (repeatedly isolated from human infections), saprobic/rock-inhabiting (repeatedly isolated from rock surfaces), or saprobic (all others, e.g., from plant leaves, cleanroom surfaces, soil crust, ant nests). The phylogenetic tree (on the left) was constructed from concatenated protein sequences of ACT1 (actin), ADE2 (phosphoribosylaminoimidazole carboxylase), H2B (histone 2B), TEF1 (translation elongation factor 1-α), and URA3 (orotidine 5′-phosphate decarboxylase). Numbers indicate predicted iron acquisition genes identified by sequence similarity, compared to *S. cerevisiae*, *A. fumigatus*, and *A. nidulans*. Gene categories—LA (low-affinity uptake): iron transporters (FetD); RIA (reductive iron assimilation, high-affinity uptake): ferroxidase-iron permease pairs (FF), ferric reductases (FRE); SIA (siderophore-mediated iron acquisition, high-affinity): biosynthetic enzymes (SidA, SidC, SidD), siderophore iron transporters (SIT), intracellular siderophore-hydrolyzing esterases (EST); MISC (miscellaneous): divalent metal transporters (SMF), heme oxygenases (HMX), vacuolar iron transporters (CCC1), ferritins (FER). For species details, genome sequences, and protein sequences, see Table S1; for *K. petricola* genes, see also Table S2.

Genes, putatively involved in iron acquisition in *K. petricola* and related black fungi (Chaetothyriales), were identified based on sequence similarity to characterized proteins from *S. cerevisiae* and *A. fumigatus*. Their distribution differs markedly between pathogenic Herpotrichiellaceae and predominantly saprobic Trichomeriaceae species (Fig. 1; Table S1). Most of the species lack a transporter for low-affinity iron uptake (FetD), but the number of ferroxidase–permease (FF) pairs correlates strongly with the lifestyle: pathogenic species such as *Fonsecaea predosoi* typically harbor at least two copies, whereas saprobic species harbor a single copy. FREs are generally more abundant in Herpotrichiellaceae. All species have at least one ortholog of *A. fumigatus* SidJ (EST), involved in intracellular iron release from siderophores, whereas SITs are markedly more abundant in Herpotrichiellaceae species. With respect to siderophore biosynthesis, all species harbor a SidC-like NRPS, but a SidD-like NRPSs and the monooxygenase SidA are absent from several Trichomeriaceae species, indicating incomplete hydroxamate siderophore pathways. However, an alternative enzyme may catalyze the formation of N⁵-hydroxyornithine. An ortholog of SidL is present, while SidF is absent in *K. petricola*. Furthermore, Herpotrichiellaceae species harbor at least two putative vacuolar iron transporters (CCC1), which are missing in most Trichomeriaceae. Interestingly, this absence correlates with the presence of a putative iron-binding ferritin (FER).

*K. petricola* possesses a single RIA complex (Fig. S1) and 12 putative FREs (Fig. S2)—more than other Trichomeriaceae, including *K. marmoricola*. Furthermore, it has four SITs, with SIT1 clustering with *S. cerevisiae* SITs and *A. fumigatus* Sit1, while the others show varying similarity to *A. fumigatus* SITs (Fig. S3). SidA- and SidD-like proteins are absent in both rock-inhabiting *Knufia* species (Fig. S4), a pattern shared with *Neophaeococcomyces aloes* and *Pasadenomyces melaninifex*, which were isolated from cleanrooms and whose natural habitats remain unknown. Similarly, the absence of vacuolar iron transporters combined with the presence of a putative ferritin (Fig. S5) is a feature shared by the three recognized rock-inhabiting species (*K. petricola*, *K. marmoricola*, and *Lithohypha guttulata*), and *N. aloes*. Additionally, the *K. petricola* genome comprises the genes encoding the conserved transcriptional regulators SRE1, HAPX, and SRB1 (Table S2).

In summary, *K. petricola* contains single-copy genes for RIA and SIA, indicating a potentially limited iron acquisition capacity. To assess the roles of the two systems under varying iron conditions, mutants were generated and characterized (Table S3).

### RIA—mediated by FTR1 and FET1—facilitates growth under iron limitations

The genes, encoding a high-affinity iron permease (FTR1) and a ferroxidase (FET1) as components of the RIA complex, are physically linked in the genome of *K. petricola* by sharing a bidirectional promoter (Fig. S1). This genomic organization is typical for fungal genomes, where one to three copies of the *ftr1-fet1* cluster are commonly found. *K. petricola* FTR1 and FET1 share 61% and 52% aa identity, respectively, with their orthologs from *A. fumigatus*, and 69% and 59% identity, and 51% and 45% identity, with those from *E. dermatitidis*.

**Fig 2:**
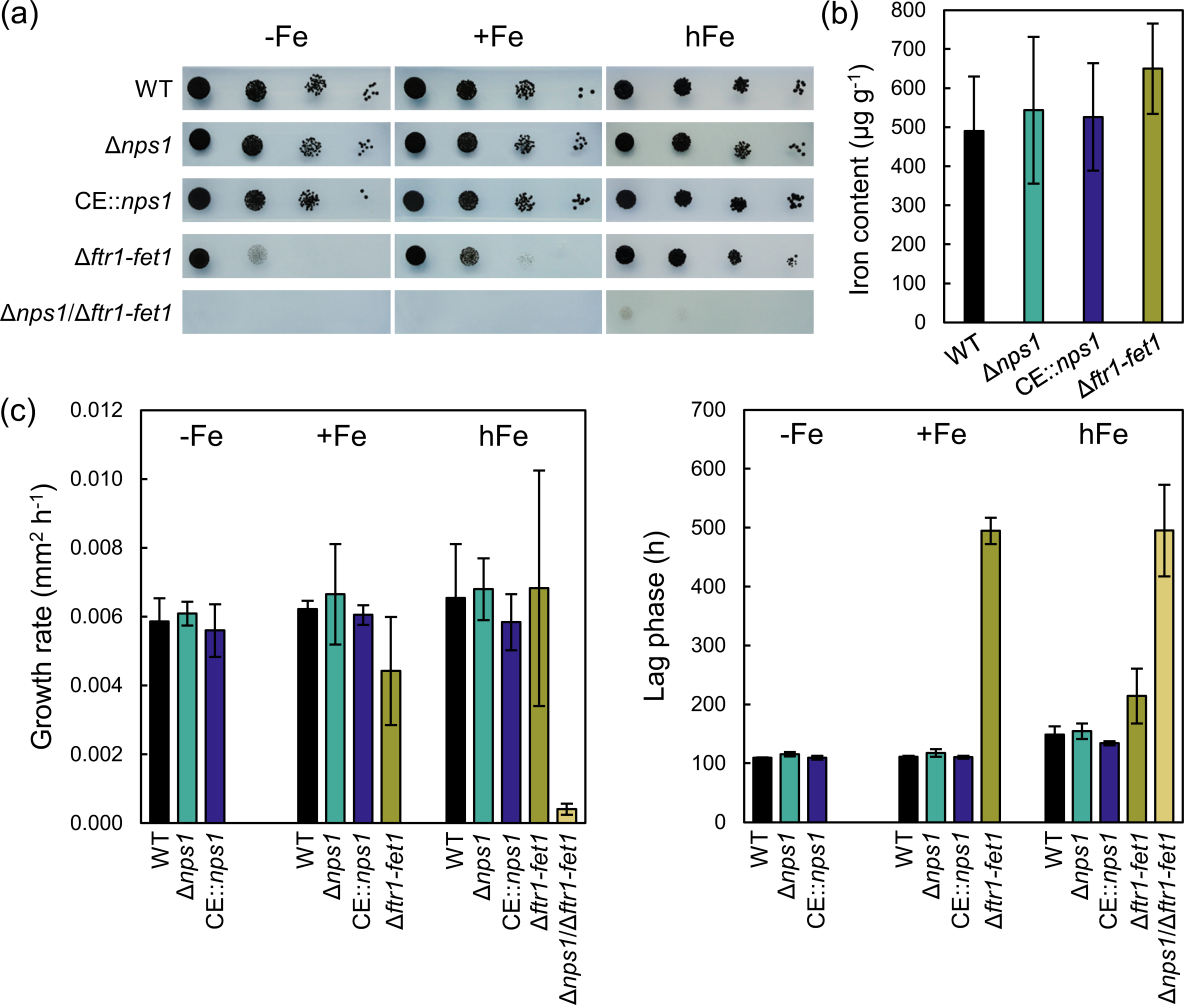
*K. petricola* acquires iron primarily through RIA by FTR1–FET1. (**a**) RIA-deficient Δ*ftr1-fet1* mutants fail to grow under iron-limited conditions. Ten microliter droplets containing 10^4^, 10^3^, 10^2^, and 10^1^ CFU of selected strains were spotted onto solid medium without iron (–Fe), or supplemented with 30 µM iron (+Fe) or 1,511 µM iron (hFe) and incubated for 12 days. (**b**) WT and iron uptake-deficient mutants accumulate comparable amounts of iron in their biomass (including cell wall). The iron content was quantified for biomass harvested from 21-day-old cultures grown on solid +Fe medium. The biomass was digested with 1 M HNO_3_ and analyzed by inductively coupled plasma-optical emission spectroscopy (ICP-OES). Shown are the averages of three independent replicates with two times the standard error or analytical uncertainty, whichever was highest. (**c**) High-iron conditions improve the growth of RIA-deficient mutants, but not to WT levels. Growth rates and lag phases on media with different iron contents were recorded by the ScanLag system. Shown are the averages of three independent replicates with two times the standard error.

To investigate the role of RIA in *K. petricola*, the genomic region encompassing both genes was deleted by replacing it with a resistance cassette, resulting in the mutant Δ*ftr1-fet1* (Fig. S6a). Growth of the generated mutants was assayed on minimal medium (MM) under three iron conditions: iron limitation (−Fe, no added iron), iron sufficiency (+Fe, 30 µM Fe), and iron repletion (hFe, 1,511 µM Fe). When cell suspensions were dropped onto agar, the Δ*ftr1-fet1* mutant exhibited considerably reduced growth on −Fe and +Fe compared to the wild type (WT), but not under hFe conditions (Fig. 2a). The quantification of the iron content in the biomass (including the cell wall) obtained from iron-limiting conditions (−Fe) yielded, however, similar results for the mutant and the WT (Fig. 2b). The growth parameters of single colonies on the three solid media were quantified using the ScanLag system, which performs time-lapse scanning of Petri dishes (Fig. 2c). The WT exhibited comparable growth rates across all tested conditions (approximately 0.006 mm^2^ h^−1^). However, its lag phase was significantly prolonged on hFe (148 ± 14 h) compared to −Fe (109 ± 1 h, *P* = 0.0001) and +Fe (111 ± 2 h, *P* = 0.0013). The Δ*ftr1-fet1* mutant did not grow sufficiently without iron to allow quantification of growth parameters. On +Fe, its growth rate was similar to that of the WT, but its lag phase was significantly extended (495 ± 22 h, *P* < 0.0001).

To verify that the observed growth phenotype of the Δ*ftr1-fet1* mutant was specifically due to the deletion of the *ftr1-fet1* locus, a complementation strain was generated by reinserting the *ftr1-fet1* pair into the neutral genomic site *igr2* (Fig. S6b). The resulting strain, Δ*ftr1-fet1*::*ftr1-fet1*, exhibited growth parameters comparable to those of the WT, including similar growth rates and lag phases under both iron-limiting and iron-sufficient conditions (Fig. S7). These results demonstrate that the growth defect of the mutant is due to the loss of *ftr1* and *fet1*, confirming their role in RIA. However, the observation that the mutant retains limited growth under low-iron conditions (−Fe and +Fe) suggests the presence of an alternative iron uptake system that partially compensates for the loss of *ftr1-fet1*.

### SIA—mediated by an extracellular, yet unidentified siderophore—requires NPS1

NPS1, comprising 4,897 aa, represents the sole NRPS of *K. petricola*. It shares 22% and 41% aa identity with SidC of *A. fumigatus* and *F. pedrosoi*, respectively, both known to participate in ferrocrocin biosynthesis (17, 33). Both Chaetothyriales NRPSs feature an additional tandem of thiolation (T) and condensation (C) domains (Fig. S4). However, ferricrocin could not be detected in either the culture supernatant or biomass of *K. petricola* (data not shown), consistent with the absence of a *sidA* ortholog (Fig. 1)—a gene needed for the production of hydroxamate siderophores such as ferricrocin (8) and typically located upstream of the NRPS-encoding gene.

To investigate whether *nps1* is involved in iron acquisition, the gene was deleted (Δ*nps1*) and its expression was elevated (Fig. S8). For the latter, the native promoter of *nps1* was replaced with the strong P*oliC* from *A. nidulans*, resulting in the strain CE::*nps1* (constitutive expression of *nps1*). To explore a potential functional interdependency between the RIA system and NPS1, the *ftr1-fet1* locus was additionally deleted in the Δ*nps1* background, generating the double mutant Δ*nps1*/Δ*ftr1-fet1*. Neither the deletion nor the constitutive expression of *nps1* resulted in any visible or quantifiable changes in colony growth under any of the three iron conditions tested (Fig. 2). This included comparable iron contents in the biomass under iron-limited conditions. In contrast, the Δ*nps1*/Δ*ftr1-fet1* mutant failed to grow on −Fe and +Fe, indicating a synthetic growth defect under low-iron conditions. On hFe, growth was partially restored, although the strain exhibited a significantly prolonged lag phase (495 ± 78 h, *P* = 0.0017) and a reduced growth rate (0.0004 ± 0.0002 mm^2^ h^−1^, *P* = 0.0252) compared to the WT.

**Fig 3:**
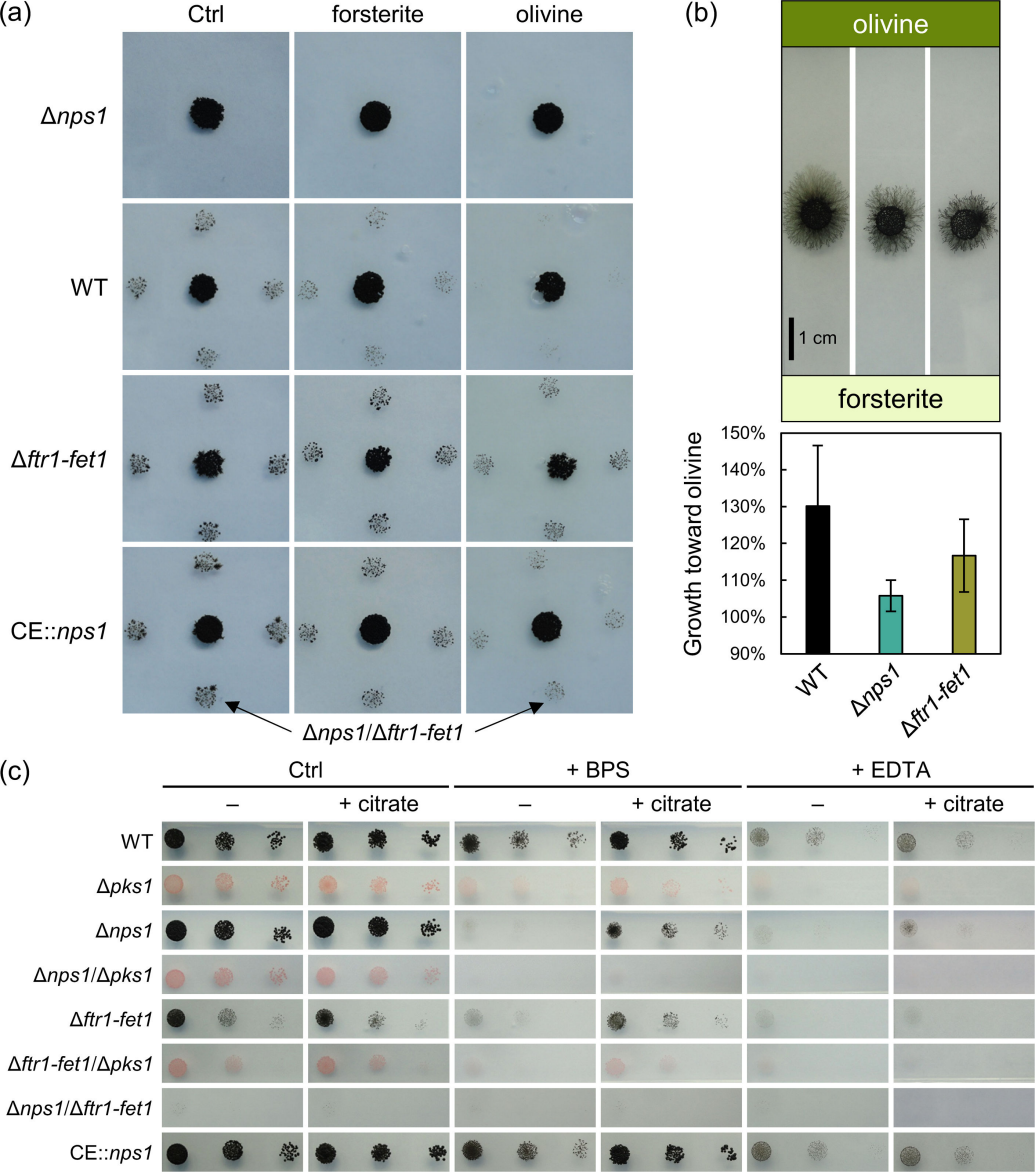
NPS1 contributes to SIA in *K. petricola*. (**a**) NPS1 is required to produce an extracellular siderophore. Four 10 µL drops each containing 10^3^ CFU of the Δ*nps1*/Δ*ftr1-fet1* mutant were spotted next to drops of Δ*nps1*, WT, Δ*ftr1-fet1,* or CE::*nps1* on solid –Fe medium alone (Ctrl) or supplemented with forsterite (Mg_2_SiO_4_) or olivine ((Mg,Fe)_2_SiO_4_). Growth of Δ*nps1*/Δ*ftr1-fet1* indicates the production of a diffusible siderophore by the neighboring strain after 34 days of incubation. (**b**) The extracellular siderophore enables chemotropism toward an iron source. Five 10 µL drops containing 10^3^ CFU of WT, Δ*nps1,* or Δ*ftr1-fet1* were spotted onto agar, which was not supplemented with iron, in between a streak of iron-rich olivine and iron-free forsterite. The relative growth rates toward olivine (growth toward olivine/growth toward forsterite, %) were calculated after 74 days of incubation. Given are the averages of three independent replicates (with five colonies each) with two times the standard error. (**c**) NPS1 is not required for growth on iron-deficient medium when RIA is operating. 10^4^, 10^3^, and 10^2^ CFU of selected strains were dropped onto MM agar without supplemented iron (–Fe) and with and without 1 mM citrate, 4.5 µM BPS, or 4.0 µM EDTA and incubated for 12 days.

Given that NPS1 appears to contribute to iron acquisition and becomes essential in the absence of a functional RIA system, we next investigated whether the NRPS might be involved in the production of a diffusible, iron-binding compound, potentially a previously unidentified siderophore. A cross-feeding assay was performed, in which the Δ*nps1*/Δ*ftr1-fet1* mutant was grown under iron-limiting conditions in proximity to either the WT, Δ*nps1*, Δ*ftr1-fet1*, or CE::*nps1*. This allowed to assess whether any of these strains could secrete an iron-chelating compound capable of rescuing the growth of the double mutant. In addition, the medium not supplemented with iron (−Fe) was supplemented with either iron-free forsterite (Mg_2_SiO_4_) or iron-containing olivine ((Mg,Fe)_2_SiO_4_) to evaluate whether mineral-derived iron could support growth under these conditions (Fig. 3a). Growth of the Δ*nps1*/Δ*ftr1-fet1* mutant was absent when co-cultivated with Δ*nps1*, but detectable next to the WT and most pronounced in proximity to Δ*ftr1-fet1* and CE::*nps1* colonies. Interestingly, similar growth of the double mutant was observed for media without and with iron-free forsterite, whereas growth was reduced in the presence of the iron-containing olivine. Together, these observations indicate that NPS1 is involved in the production of an extracellular, diffusible siderophore. The reduced growth stimulation in the presence of olivine suggests lower siderophore production. In an alternative experiment, olivine and forsterite were used to investigate whether WT, Δ*nps1*, and Δ*ftr1-fet1* can actively grow toward an iron source, as an indication of chemotropism (Fig. 3b). The strains were inoculated between the two minerals on −Fe medium without a supplemented nitrogen source to promote filamentous growth. This revealed that the Δ*nps1* mutant did show significantly (*P* = 0.0280) reduced growth toward the olivine than the WT. The minimal filamentous growth of the Δ*ftr1-fet1* mutant prevented the quantification of its chemotropism. These results imply that the unknown siderophore produced by *K. petricola* likely facilitates iron acquisition from olivine.

To confirm the growth phenotypes of the Δ*nps1* mutant, a complementation strain was constructed by reinserting *nps1* into its native locus through the *in vivo* assembly of the large coding region from four overlapping amplicons (Fig. S8). Restoration of siderophore production in the Δ*nps1*::*nps1* strain was demonstrated by its ability to support growth of the adjacent Δ*nps1*/Δ*ftr1-fet1* mutant (Fig. S9). In sum, the data demonstrate that a SIA system operates in *K. petricola*, involving an extracellular, yet unidentified siderophore.

### *K. petricola* and other rock inhabitants exhibit high sensitivity to iron depletion

The addition of chelators to the culture medium intensifies iron limitation by binding residual trace iron, even in media considered iron-free. Under such controlled conditions, both the iron-binding strength of metabolites, such as siderophores, and the total iron-binding strength of organisms can be experimentally assessed and compared.

When medium not supplemented with iron (−Fe) was supplemented with 4.5 µM of the ferrous iron chelator bathophenanthroline disulfonate (BPS) or 4.0 µM of the ferric iron chelator ethylenediaminetetraacetic acid (EDTA) (Fig. 3c), all tested strains exhibited reduced growth: WT and CE::*nps1* showed comparable reduced growth, while growth of both Δ*nps1* and Δ*ftr1-fet1* was nearly abolished. Notably, the growth inhibition of Δ*nps1* was more pronounced, considering that this mutant displayed more robust growth on −Fe medium without chelators compared to Δ*ftr1-fet1*, indicating that RIA is more sensitive toward BPS, as observed in *A. fumigatus* by Schrettl et al. (15). The complemented strain Δ*nps1*::*nps1* exhibited BPS sensitivity comparable to that of the WT (Fig. S9). Interestingly, supplementation with 1 mM citrate enabled the Δ*nps1* mutant to grow in the presence of BPS and EDTA to nearly the same extent as the WT (Fig. 3c), suggesting that citrate may partially compensate for the loss of siderophore function. The Δ*ftr1-fet1* mutant also showed enhanced growth upon citrate addition. However, no improvement was observed for the double mutant Δ*nps1*/Δ*ftr1-fet1*, indicating that both iron acquisition pathways are required for citrate-supported growth under chelator stress. Note that, even though the pH of the medium was buffered, citrate supplementation might as well have caused these effects through the reduction of the pH.

**Fig 4:**
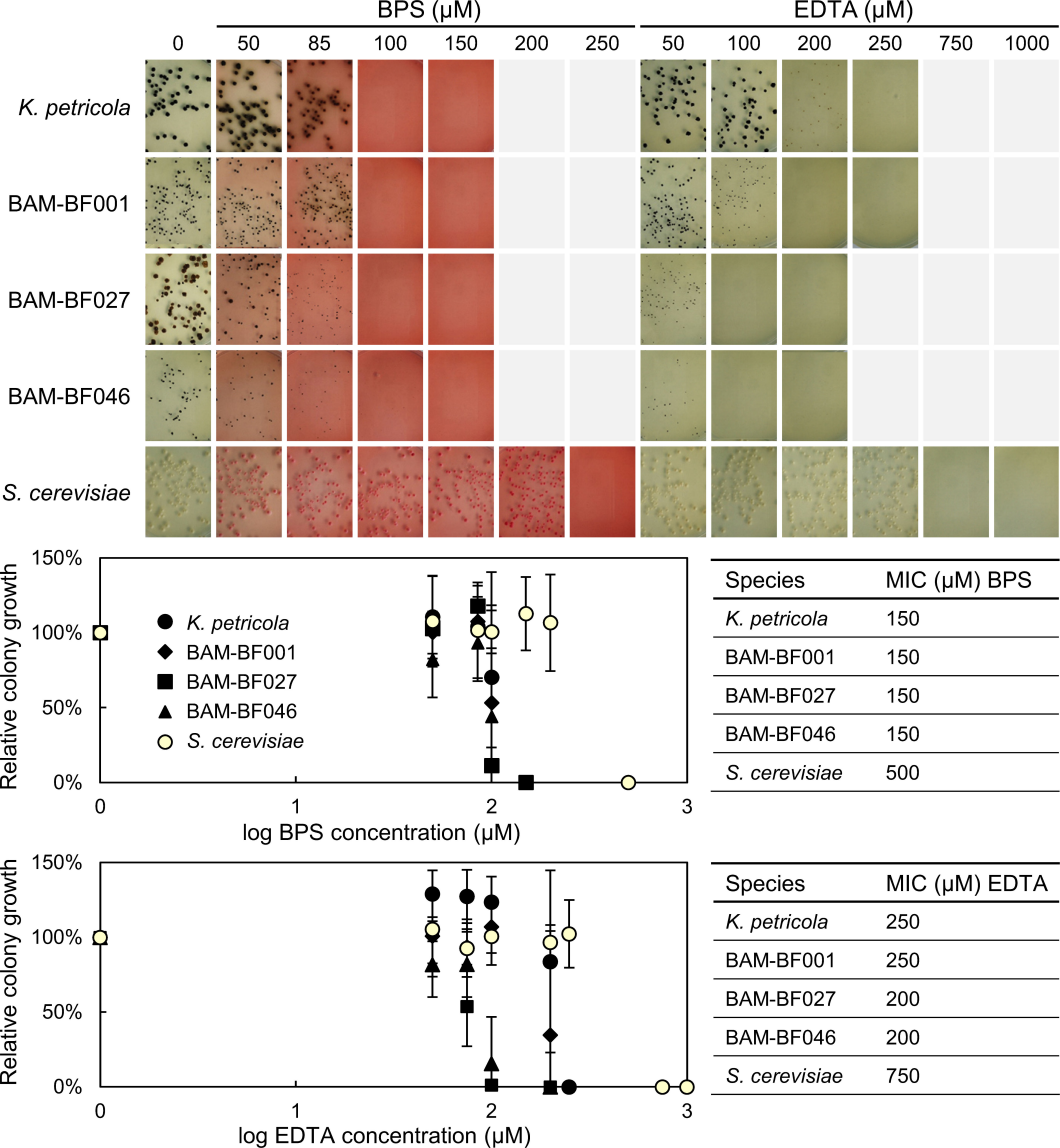
*K. petricola* and other rock-inhabiting black fungi are more sensitive toward iron chelators than *S. cerevisiae*. In all, 200–450 CFU of *K. petricola* WT (A95), rock-inhabiting fungi (BAM-BF001, BAM-BF027, and BAM-BF046), and *S. cerevisiae* DSM 1333 were distributed on MEA, which was buffered at pH 6 and contained BPS or EDTA in varying concentrations. After 14 days of incubation, images were taken, and the visible colonies were counted for calculating relative colony growth in percent (number of colonies on supplemented MEA/number of colonies on MEA without chelator × 100) of each replicate. Shown are the averages of three independent replicates with two times the standard error. The MIC of BPS and EDTA was defined as the lowest concentration at which the relative colony growth was 0%.

To assess whether other rock-inhabiting black fungi are similarly sensitive to iron chelators as *K. petricola*, three species isolated from solar panels and belonging to different classes were tested: BAM-BF001 (Eurotiomycetes), BAM-BF027 (Arthoniomycetes), and BAM-BF046 (Dothideomycetes). As a reference, *S. cerevisiae* was included, which is known to thrive under iron-limited conditions (34). The minimum inhibitory concentrations (MICs) of the two iron chelators were determined by inoculating malt extract agar (MEA) with varying BPS and EDTA concentrations with CFU suspensions and quantifying colony formation after 2 weeks of incubation. All rock-inhabiting black fungi exhibited comparable sensitivities to these chelators with MICs of 150 µM for BPS and 200–250 µM for EDTA. By contrast, *S. cerevisiae* was less sensitive, with MICs of 500 µM for BPS and 750 µM for EDTA (Fig. 4).

Taken together, iron depletion by adding iron chelators to the medium (further) reduced the growth of RIA- and SIA-deficient *K. petricola* mutants, suggesting that the NPS1-derived siderophore is either weak or produced in low amounts. This growth inhibition could be partially reversed by adding citrate, demonstrating that iron-citrate is an iron source. Moreover, *K. petricola* shares the sensitivity to iron chelators with other unrelated rock-inhabiting black fungi.

### Melanin adsorbs and reduces iron, but is not involved in iron acquisition

To investigate the role of melanin and its metal-binding properties in iron acquisition, melanin-deficient RIA and SIA mutants were generated. For this, *pks1*, encoding the polyketide synthase essential for DHN melanin synthesis, was deleted in Δ*nps1* and Δ*ftr1-fet1* backgrounds (Fig. S10), and the resulting double mutants were compared with the WT and the Δ*pks1* mutant (30).

**Fig 5:**
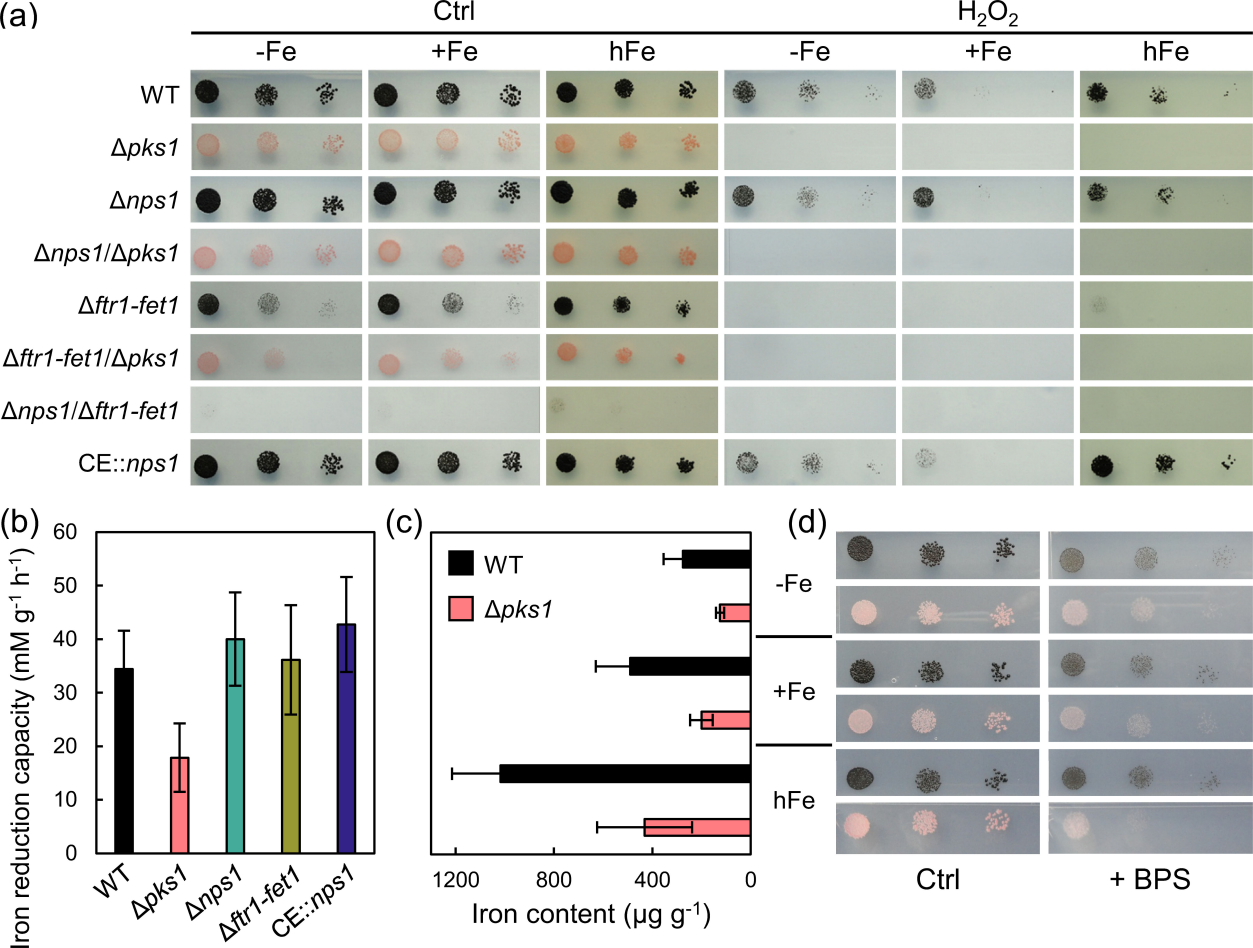
The melanin of *K. petricola* facilitates iron reduction and adsorption but is not involved in iron uptake. (**a**) Non-melanized mutants exhibit increased sensitivity to oxidative stress induced by hydrogen peroxide (H_2_O_2_)—independent of the iron content. CFUs of melanized and non-melanized Δ*pks1* strains were point-inoculated on –Fe, +Fe, and hFe agar without and with 2 mM (–Fe and +Fe) or 2.75 mM (hFe) H_2_O_2_, and incubated for 12 days. The −Fe controls in panel a are identical to controls in Fig. 3c because they derive from the same parallel experiment. (**b**) The non-melanized Δ*pks1* mutant, in contrast to RIA- and SIA-deficient strains, exhibits attenuated iron-reducing activity. Liquid –Fe medium was inoculated with 10^6^ CFU of the indicated strains and incubated for 14 days. The iron reduction capacity—expressed as mM Fe(II) g^−1^ biomass h^−1^—was determined by measuring the absorbance at 535 nm (A_535_) of the cultures after incubation with the iron-chelator BPS. Shown are the averages of four independent replicates with two times the standard error. (**c**) The biomass of the non-melanized Δ*pks1* mutant contains less iron compared to that of the WT. The two strains were cultivated for 21 days on solid –Fe, +Fe, and hFe medium. Iron contents—expressed as µg iron per g biomass—were quantified via ICP-OES analyses of the digested biomass. Shown are the averages of three independent replicates with two times the standard error or analytical uncertainty, whichever was highest. (**d**) The melanized WT (black) and the non-melanized Δ*pks1* mutant (pink) exhibit similar growth on iron-depleted conditions, regardless of the iron availability during precultivation. The two strains were cultivated for 7 days on agar without iron (–Fe) or with iron (+Fe, or hFe). Cells from these cultures were then spotted onto –Fe agar supplemented with 4.5 µM BPS as indicated. Images were taken after 12 days of incubation.

In a drop assay, the sensitivity of the strains to oxidative stress induced by hydrogen peroxide (H_2_O_2_) under different iron conditions was evaluated. All strains showed reduced growth with H_2_O_2_: Δ*nps1* and CE::*nps1* were equally sensitive to H_2_O_2_ as the WT, whereas all other strains failed to grow with H_2_O_2_, highlighting that Δ*pks1* and Δ*pks1*/Δ*nps1* mutants are more sensitive to H_2_O_2_ compared to their melanized counterparts (WT, Δ*nps1*) (Fig. 5a). The dark pigmentation and the tolerance to H_2_O_2_ were found restored in the control strain Δ*pks1*::*pks1*, generated in this study by introducing *pks1* with its native regulatory regions into the *igr2* locus of Δ*pks1* (Fig. S10).

The iron-reducing capacity was assessed in both the culture supernatants and biomass of WT, Δ*pks1,* and the melanized RIA- and SIA-deficient mutants. The biomass of the Δ*pks1* mutant exhibited a significantly lower iron-reducing capacity compared to the other strains (e.g., 53% of WT, *P* = 0.045), which all had similar reductase activities (Fig. 5b). In all strains, the culture supernatants displayed substantially higher iron-reducing capacities with no significant differences between strains (Fig. S11b). These findings demonstrate that cell-wall-bound melanin can reduce ferric iron, while extracellular components dominate iron reduction in the supernatant. Furthermore, the iron content of the biomass—an indication of iron adsorption—was measured for the melanized WT and the non-melanized Δ*pks1* mutant after cultivation under different iron concentrations. Across all conditions, the Δ*pks1* mutant consistently showed significantly lower iron levels in its biomass compared to the WT: 45% (*P* = 0.0039) on −Fe, 41% (*P* = 0.0018) on +Fe, and 42% (*P* = 0.0071) on hFe (Fig. 5c).

To investigate whether the iron adsorbed to melanin could serve as an extracellular iron reservoir or facilitate iron sequestration from the environment, another drop assay was performed. WT and Δ*pks1* strains were precultured under −Fe, +Fe, and hFe conditions. Subsequently, cell suspensions were spotted onto −Fe agar with and without 4.5 µM BPS. Both strains exhibited comparable growth, regardless of the iron conditions during preculture.

Finally, to determine whether melanin formation influences growth in the presence of iron chelators, sensitivity assays were performed across different genetic backgrounds (WT, Δ*nps1*, and Δ*ftr1-fet1*) with BPS and EDTA, with and without citrate supplementation (Fig. 3c). The deletion of *pks1* did not seemingly alter the sensitivity of the strains to iron chelators, indicating that melanin does not play a detectable role in chelator resistance under the tested conditions.

## DISCUSSION

Iron is an indispensable micronutrient for all living organisms, driving intense competition for its acquisition. This competition is particularly evident in parasitic interactions, where hosts actively restrict iron availability to inhibit the growth of fungal invaders. Saprobic fungi, on the other hand, acquire iron by digesting dead organic material and may protect their nutrient sources from competitors through efficient iron uptake, rapid growth, and the production of toxic secondary metabolites. In contrast, mutualistic relationships—such as those found in lichens—are characterized by cooperation, where different organisms harmonize their nutrient acquisition strategies.

We hypothesized that iron uptake by rock-inhabiting black fungi is unconventional, resulting in a high sensitivity to iron chelators. These organisms (i) are considered free-living, that is, they do not rely on interaction partners, although they may exist within microbial communities (25), (ii) colonize oligotrophic substrates exposed to sunlight, which imposes UV radiation, temperature fluctuations, and desiccation stress—conditions that limit the growth of fast-growing saprobes, (iii) constitutively produce melanin, a pigment with known iron-adsorbing properties (26, 35), and (iv) may obtain iron from the weathering of iron-containing rock substrates (36). Overall, black fungi inhabiting such extreme environments are likely not exposed to immediate competition for iron, which may have enabled the evolution of simplified and more energy-efficient iron acquisition and storage strategies.

To investigate this hypothesis, we first employed a bioinformatics approach, aiming to compare the potential iron acquisition strategies of selected Chaetothyriales black fungi—including pathogenic, saprobic, and rock-inhabiting species—based on their genomic repertoires. The results demonstrate that the closely related rock inhabitants *K. petricola* and *K. marmoricola*, both repeatedly isolated from marble surfaces (37, 38), possess a reduced set of iron acquisition genes. This gene set is characterized by a single RIA complex, the absence of key siderophore biosynthesis enzymes (*sidA*, *sidD*), the lack of a vacuolar iron transporter, and the presence of a ferritin, which may facilitate intracellular iron storage, as in other eukaryotes (39, 40). In contrast, *K. obscura*, isolated, for example, from a cleanroom facility or a gasoline tank (41), contains a slightly altered gene set. These differences may reflect ecological adaptation and could contribute to future refinement of the genus *Knufia*. The closely related pathogenic species *A. fulminans* possesses a second RIA complex, additional SITs, and a putative vacuolar iron transporter. This pattern is consistent with that of pathogenic Herpotrichiellaceae species, which typically contain at least two RIA complexes, a higher number of SITs, the complete set for SIA (*sidA*, *sidC*, and *sidD*), and multiple CCC-type transporters. Although non-pathogenic black fungi possess fewer putative SITs and FREs than their pathogenic relatives, the presence of multiple copies—combined with the siderophore specificity of these transporters and reductases (8)—suggests that they are equipped to utilize a range of siderophores, albeit a narrower spectrum than pathogenic species. The ability to utilize xenosiderophores, including those not originating from fungi, provides a competitive advantage by conserving metabolic energy (42–45). In addition, black fungi may retrieve iron from the degradation of self-produced or foreign heme via the heme oxygenase (HMX1). Uptake of xenosiderophores or heme may occur incidentally when these compounds are encountered in the environment; however, the likelihood of acquiring them from living or dying microorganisms is considerably higher within subaerial biofilms. Through this analysis, we identified distinct patterns of iron acquisition genes in pathogenic versus non-pathogenic black fungal species. These findings will benefit from the availability of additional genome sequences of black fungi from diverse habitats, which are expected to emerge from the community science project STRES (https://stresblackfungi.org/) (46). With a broader data set and verified ecological or physiological traits, it may eventually become possible to predict the pathogenic/opportunistic potential of black fungi based on their iron acquisition gene profiles.

**Fig 6:**
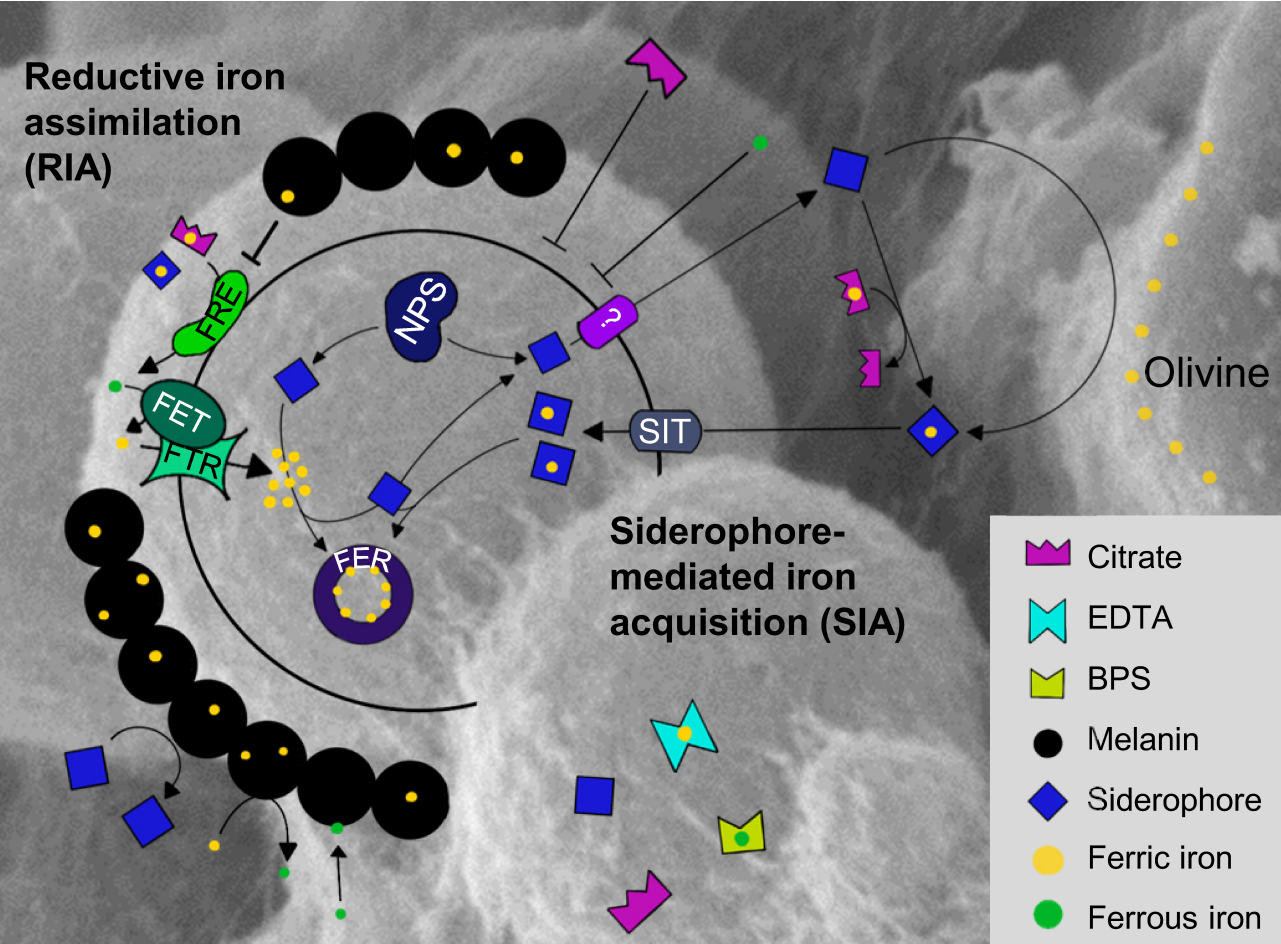
Proposed model of iron acquisition to intracellular storage in *K. petricola*. Iron can be taken up via a SIA system consisting of (xeno)siderophores and siderophore transporters (SIT1-4), but is primarily taken up via the RIA system consisting of ferric reductases (FRE1-12), a ferroxidase (FET1), and an iron permease (FTR1). The siderophore synthesized by the non-ribosomal peptide synthetase NPS1 remains unidentified and appears ineffective, making RIA the dominant mechanism and increasing the susceptibility to strong iron chelators such as BPS and EDTA. Direct uptake of iron-bound citrate is not feasible; however, citrate-bound iron can be transferred to the RIA system or to the siderophore. Melanin, although adsorbing and reducing iron, does not contribute to iron uptake or storage as iron bound to melanin is not available for growth. Intracellular iron is likely stored in ferritin (FER1), as proteins for vacuolar iron storage are absent.

Experimentally, the functionality of RIA and SIA systems in *K. petricola* was demonstrated (Fig. 6). Under laboratory conditions simulating the free-living lifestyle, *K. petricola* primarily acquires iron via the RIA system, encoded by *ftr1* and *fet1*, and secondarily through the SIA pathway involving *nps1*. Mutants lacking both *ftr1-fet1* and *nps1* are only viable on medium supplemented with high amounts of iron (hFe), indicating that RIA and SIA are the major relevant mechanisms in *K. petricola*—at least under laboratory conditions. Cross-feeding assays with the Δ*nps1*/Δ*ftr1-fet1* mutant as test strain demonstrated the presence of a secreted and diffusible siderophore in *K. petricola*, corroborating previous findings from a chrome azurol S assay in this species (47). Biosynthesis of the siderophore requires the sole SidC-like NRPS, NPS1. However, high-performance liquid chromatography-tandem mass spectrometry did not allow us to detect distinct masses present in the extracts of the WT and CE::*nps1* and absent in those of Δ*nps1*. Masses corresponding to ferrichrome-type siderophores like ferricrocin could also not be detected. Therefore, future work should aim to elucidate the nature of this compound.

Iron is present at the surface of olivine either in the form of ferric (oxyhydr)oxide precipitates or as ferric iron within the structure of olivine (36). Whether the siderophore can accept iron from the former or the latter is unknown. Such a chemotropic response is, however, particularly remarkable, given that siderophore production was reduced in the presence of iron-rich olivine, suggesting that even low levels of the siderophore are sufficient to facilitate iron acquisition under these conditions. A lower siderophore production in the presence of an iron source was also observed for *A. fumigatus* (48) and *A. nidulans* (49), in which genes involved in both RIA and SIA are repressed by the GATA-transcription factor SreA under high iron, while the bZIP transcription factor HapX represses iron-consuming pathways and activates siderophore production under iron limitation (50–52). Assuming a similar regulatory network for RIA and SIA genes in *K. petricola*, potentially governed by conserved transcriptional regulators, the disruption of RIA via Δ*ftr1-fet1* or SIA via Δ*nps1* may mimic iron-limiting conditions. This could, in turn, trigger compensatory upregulation of the remaining iron acquisition pathway, thereby enabling continued growth despite the loss of one system.

The addition of citrate alleviated the iron limitation of *K. petricola* under iron-depleted conditions caused by chelators. The lack of improved growth of the Δ*nps1*/Δ*ftr1-fet1* mutant under iron-limiting conditions with citrate addition suggests that direct uptake of citrate-bound iron is unlikely. This aligns with observations in *Ustilago sphaerogena,* where iron, but not citrate, was taken up when iron citrate was provided (53). The restored growth of both Δ*ftr1-fet1* and Δ*nps1* upon citrate addition suggests that both the FREs of RIA and the siderophore of *K. petricola* can mobilize iron from citrate. The ability of the RIA system to utilize citrate-bound iron has also been demonstrated in *S. cerevisiae*, which lacks its own siderophores (54) but can grow on ferric citrate as an iron source (55). Additionally, siderophores have been shown to acquire iron from ferric oxides via low-molecular-weight acids like citrate (56, 57), supporting the idea of iron handover from citrate to siderophores.

We demonstrated that the cell-wall-bound melanin of *K. petricola* is capable of adsorbing and reducing iron. Iron adsorption capacity, inferred from the iron content of the biomass, correlated with iron availability in the culture medium for both the WT and the Δ*pks1* mutant. However, across all conditions, the non-melanized Δ*pks1* mutant consistently showed lower iron accumulation compared to the melanized WT, demonstrating that melanin adsorbs iron. This finding aligns with earlier studies reporting that melanized fungal biomass tends to accumulate more metals like iron than albino biomass, and that fungal melanin itself could adsorb metals (35, 58–60). Similarly, the higher iron-reducing capacity observed for the melanized biomass of *K. petricola* (WT) compared to the non-melanized biomass (Δ*pks1*) is consistent with previous reports on the iron-reducing properties of enzymatically produced DOPA (3,4-dihydroxyphenylalanine) melanin (61), autoxidized DOPA melanin (62), and DOPA melanin-producing *Cryptococcus neoformans* (63). This reduction is likely mediated by reduced catechol compounds of the melanin, which are hydrophilic and therefore positioned at the surface, facilitating interaction with iron ions (60). The observation that the higher iron content of the WT inoculants compared to that of the Δ*pks1* inoculants did not translate into a reduced sensitivity toward BPS suggests that the additional melanin-bound iron in the WT was not readily available for supporting the growth of new cells. Similarly, and in contrast to other fungi—where disruption of iron uptake pathways typically results in both reduced cellular iron contents and impaired growth under low-iron conditions (17, 54, 64, 65)—the growth defect of the (melanized) Δ*ftr1-fet1* mutant on iron-limited media did not correlate with a decrease in total iron content. These findings further support the notion that iron adsorbed to melanin cannot be mobilized to sustain growth. Considering that melanin can compete with chelators such as citrate, adenosine 5′-diphosphate (ADP), and possibly EDTA for both ferrous and ferric iron (60, 62), this lack of bioavailability is reasonable. The observation that melanized and non-melanized strains exhibited similar sensitivity to iron depletion caused by chelators suggests that melanin does not sequester iron in a way that significantly interferes with either RIA or SIA. Apparently, the kinetics of both high-affinity iron uptake systems outcompete the iron adsorption of melanin. Taken together, these observations indicate that, while melanin of *K. petricola* can adsorb and reduce iron, it neither impairs nor enhances iron acquisition via RIA or SIA. Instead, melanin appears to function as a passive iron sink, adsorbing lingering iron, making it no longer accessible to both uptake systems—and unable to react with H_2_O_2_ to generate reactive oxygen species (ROS) via the Fenton reaction (66, 67). The ability of melanin to bind and stabilize iron in a non-reactive form likely contributes to cellular protection, providing an explanation for why the non-melanized *K. petricola* strains were found to be more sensitive toward oxidative stress than their melanized counterparts.

The addition of chelators to the cultivation medium mimics the natural competition for chelated iron, where only organisms equipped with strong siderophores can mobilize iron from weaker chelators. In *K. petricola*, growth inhibition by the chelators BPS and EDTA in drop assays using iron-free MM began at concentrations as low as 4.5 µM and 4.0 µM, respectively. MICs, determined based on colony formation from plated CFU on MEA, were 150 µM for BPS and 200–250 µM for EDTA. These MIC values are markedly lower than those observed for *S. cerevisiae* under identical experimental conditions, indicating *K. petricola* and the other rock-inhabiting fungi are more sensitive to iron chelation than *S. cerevisiae*. This increased sensitivity implies that these fungi possess insufficient iron to sustain growth, potentially because of restricted iron acquisition and/or storage. To facilitate comparisons, we compiled MIC data for BPS and EDTA across various fungal species (Table S4), acknowledging that MIC values are often difficult to compare between studies due to variations in media composition, experimental setups, and particularly inoculum size—as highlighted by Kubo et al. (68). This overview reveals that *K. petricola* and related rock-inhabitants exhibit MICs for these strong iron chelators that are one to two orders of magnitude lower than those reported for other fungi.

The unusually low MICs of BPS and EDTA observed for rock-inhabiting fungi may result from a combination of factors. Among these, siderophore production stands out as the most compelling explanation: siderophore-deficient strains of *K. petricola* and other fungi (17, 69) consistently show higher sensitivity to iron chelators compared to their respective WTs. Despite the observed properties of the *K. petricola* siderophore—such as its secretion, diffusion through the agar, allowing (limited) iron acquisition from BPS and olivine, uptake by neighboring cells, and driving chemotropic behavior—the low MIC values of *K. petricola* indicate that it is relatively ineffective in acquiring iron under high chelator conditions. Typically, siderophores bind ferric iron with very high affinity, exhibiting binding constants in the range of 10^25^ and 10^62^ (70). For comparison, BPS binds ferrous iron with a binding constant of 10^21^ (71), while EDTA binds ferric iron with a binding constant of 10^25^ (72). In addition to the possibility that the *K. petricola* siderophore has a relatively low affinity for iron, its limited effectiveness may also be due to low production levels or a predominant localization in the cytosol or at the plasma membrane. Especially in oligotrophic environments, an amphiphilic siderophore that remains predominantly associated with the plasma membrane and only occasionally diffuses into the extracellular space may offer a distinct advantage (73). By retaining the siderophore at the cell surface, the rock-inhabiting fungi may reduce their loss to the surroundings and thereby save metabolic energy that would otherwise be lost by continuous siderophore synthesis. To date, amphiphilic (coprogen) siderophores have been reported from one fungal species, *Trichoderma hypoxylon* (74).

Siderophore production does, however, not necessarily correlate with higher MIC values for EDTA and BPS. For example, the yeasts *Candida* species and *S. cerevisiae* exhibit relatively high MICs despite their inability to produce siderophores—values that exceed those observed for the tested black fungi (Table S4). However, melanin production as a shared trait among the four rock-inhabiting fungi is not the underlying reason. Instead, the relatively high MIC values in *S. cerevisiae*, indicating higher tolerance to iron deprivation, may be explained by efficient iron storage, particularly the sequestration of iron in vacuoles mediated through CCC1 (75) and its remobilization by an additional RIA (FET5–FTH1) complex (76). As genes for vacuolar iron storage are absent from *K. petricola* and *K. marmoricola*, it is plausible that their limited tolerance to iron-depleted conditions (reflected in low MIC values) results also from insufficient intracellular iron storage. This deficiency may not be sufficiently compensated for by the ferritin, assuming the gene is expressed and produces a functional iron-binding protein. A last explanation, of whose impact we can only speculate, would be given by the natural environment of rock-inhabiting fungi. Most fungal species whose iron uptake was studied to date are pathogens that tend to inhabit environments with higher nutrient concentrations but more competition, thus having a rather copiotrophic lifestyle (77). Perhaps such different lifestyles also come with differences in their regulation of nutrient uptake, as reported for bacteria (78).

Irrespective of the underlying reason, the low MICs of *K. petricola* and other rock-inhabiting fungi open avenues for the use of strong iron chelators in a mitigation strategy to prevent material colonization. These fungi not only colonize stone surfaces of culturally significant monuments (79) but also energy-generating photovoltaic cells (27, 80, 81). Together with other organisms and abiogenic deposits, rock-inhabiting fungi block solar radiation, having been shown to lower the efficiency of the solar panels by up to 11% after 18 months (82). Removing or preventing the formation of the typical microcolonies of these fungi has proven challenging (29). Considering the urgent need to derive more fossil fuel-free energy (83), a novel approach using biocides would therefore be most welcome. EDTA, although not considered fungicidal or only inhibitory at higher concentrations (84, 85), prevented the growth of rock-inhabiting species at relatively low concentrations (i.e., acts fungistatic). The EU Risk Assessment Report for EDTA (86) states that one should limit the risks due to the high emissions in an industrial context, leading to risks for aquatic organisms. The application of EDTA on plant leaves as a fertilizer (assuming the worst-case approach) is not considered a risk for terrestrial organisms (86), even though the chelated form is commonly applied with concentrations as high as 2.5 mM (87). Nevertheless, care should be taken to use chelators which are more easily degradable than EDTA such as N,N’-ethylenediaminedisuccinic acid (EDDS) (88–90), iminodisuccinic acid (IDS) (89, 90), or 2-((1,2-dicarboxyethyl)amino)pentanedioic acid (91). To exert a fungistatic effect, these chelators still need to be sufficiently strong. IDS has a binding constant for ferric iron of 10^14^ (92), only marginally higher than the binding constant of citrate for ferric iron of 10^12^ (93), and will thus not be useful. EDDS, however, has a binding constant of 10^22^ (94), comparable to EDTA (*K* = 10^25^) (72), and is therefore of interest within this context. Future work should focus on evaluating chelator-based approaches under real-world conditions to develop sustainable strategies for preventing fungal growth on exposed surfaces.

## MATERIALS AND METHODS

### Fungal culturing and reagents

*K. petricola* strain A95, isolated from a marble stone surface near the Philopappos monument in Athens, Greece (95), was used as WT (Table S3). Other rock-inhabiting black fungi, that is, BAM-BF001 (Eurotiomycetes), BAM-BF027 (Arthoniomycetes), and BAM-BF046 (Dothideomycetes) were isolated from solar panels in Berlin, Germany (unpublished). *S. cerevisiae* strains used were DSM 1333 and FY843 (96). Fungal cultures were maintained on malt extract agar (MEA), containing 20.0 g L^−1^ glucose, 0.1 g L^−1^ casein peptone, 20.0 g L^−1^ malt extract, and 20.0 g L^−1^ Kobe agar. Unless stated otherwise, experiments were conducted with minimal medium (MM) containing 58.4 mM D-sucrose, 35.3 mM NaNO_3_, 5.878 mM H_2_KPO_4_, 6.707 mM KCl, 2.029 mM MgSO_4_*7H_2_O, 10.0 µM CaCl_2_*2H_2_O, 1.002 µM ZnSO_4_*7H_2_O, 1.001 µM MnCl_2_, 1.000 µM CuSO_4_*5H_2_O, 1.00 µM H_3_BO_3_, 0.101 µM CoCl_2_*6H_2_O, 0.0992 µM Na_2_MoO_4_*2H_2_O, 0.023 µM Na_2_SeO_3_*5H_2_O, 0.101 µM NiCl_2_*6H_2_O, and 15.0 g L^−1^ bacteriology grade agar, adding no (−Fe), 30 µM (+Fe), or 1,511 µM (hFe) FeSO_4_*7H_2_O. All glassware was cleaned overnight with 1 M HNO_3_ to prevent any chemical contamination. Media were buffered at pH 6 with 12.30 mM 2-(N-morpholino)ethanesulfonic acid (MES), added after autoclaving. Cultures were incubated in the dark at 25°C. Biomass of all strains was disaggregated by glass beads (3–4 mm diameter) in a Retsch mixer mill (10 min at 30 Hz), and washed twice with sterile MilliQ water. Numbers of CFU, that is, single cells and cell aggregates, were determined using a Thoma cell counting chamber.

### Growth assays

All strains were precultured for 7 days on –Fe agar, except the Δ*nps1*/Δ*ftr1-fet1* mutant, which was precultured on hFe agar to promote growth. Δ*nps1* mutants were never grown in the same Petri dish as *nps1*-expressing strains to avoid cross-feeding by other strains. Growth on different solid media was tested by semi-quantitative drop assays. For this, 10 µL of serial dilutions with 10^6^, 10^5^, 10^4^, and (in certain cases) 10^3^ CFU mL^−1^ were dropped onto solid medium with or without Fe. Varying concentrations of iron chelators BPS, Na_2_EDTA*2H_2_O, or citrate) were added after autoclaving. For inducing oxidative stress, H_2_O_2_ was added to the medium after autoclaving. To test the use of melanin as an iron source, WT and Δ*pks1* were grown for 7 days on –Fe, +Fe, or hFe agar covered with cellophane, harvested and treated as described before, and point-inoculated on –Fe with and without 4.5 µM BPS. For the cross-feeding assay, –Fe agar, without additive or supplemented with 2.5 mg mL^−1^ synthetic forsterite or 2.5 mg mL^−1^ natural iron-containing olivine (India, Hausen Mineraliengrosshandel, both ground to < 63 µm), was inoculated with four 10 µL drops containing 10^3^ CFU of Δ*nps1*/Δ*ftr1-fet1* in between of which (and with a distance of 20 mm) one 10 µL drop (10^3^ CFU) of the test strain was dropped. To test whether strains can grow toward an iron source, 50 mg of the same ground olivine and forsterite was streaked out on opposite sides of rectangular Petri dishes containing –Fe agar. Exactly in between the mineral streaks, at a distance of 50 mm, 10 µL drops containing 10^3^ CFU of the test strains were spotted in a row. After 74 days, images were taken, and the distances of growth from the initial drop toward olivine and forsterite were quantified. The ratio of growth toward olivine vs. toward forsterite (i.e., the relative growth toward olivine) was calculated for all five colonies of each Petri dish, and the average was taken. Each growth assay was conducted at least three times with reproducible outcomes. Representative images from a single experiment are presented.

The lag phases and growth rates of individual colonies were determined using the ScanLag system (97). For this, –Fe, +Fe, and hFe agar were inoculated with 500 CFU of the respective strains. The Petri dishes (9 cm in diameter) were subsequently incubated in flatbed scanners (V600, Epson, connected to a computer running Windows 10 OS) in the dark at 25°C. Scans were taken every 6 h using a custom PowerShell script. The lag phase and growth rate were quantified using a custom Matlab script based on the Matlab code of Levin-Reisman et al. (97), assuming linear growth. As a slight inhibition effect was observed (i.e., a decrease in the growth rate with an increasing CFU, data not shown), only Petri dishes with a CFU in the range of 50–200 (except for Δ*nps1*/Δ*ftr1-fet1* which had CFUs in the range of 6–23, which might have caused an overestimation of its growth rate) were analyzed.

To define the MIC of EDTA and BPS, 20 mL MEA per Petri dish (9 cm), buffered at pH 6 with 12.30 mM MES and supplemented with different concentrations of EDTA or BPS, was inoculated with 200–450 CFU (three independent replicates were run per condition) and incubated for 14 days in the dark. Relative colony growth in percent was calculated.

### Bioinformatics analyses

Analyses were performed using Geneious Prime 2024.0.7 (Biomatters Ltd). Genome assemblies of selected species were retrieved from GenBank (Table S1). *De novo* gene prediction and revision of incorrect gene models were performed using the Augustus plugin 0.1.1 (98). Candidate proteins were identified by internal BLAST searches (default parameters) using sequences from *S. cerevisiae* and *A. fumigatus* as queries. Conserved protein domains were identified using the InterProScan plugin 2.1.0 (99). Sequences of the *K. petricola* iron acquisition genes have been submitted to GenBank (Table S2). All protein sequences used for analyses in Fig. 1 are listed in Table S1. Protein sequences were aligned using MUSCLE v5.1 with default parameters. Phylogenetic trees were inferred with the PHYML plugin 2.2.4 (100), applying the LG amino acid substitution model and 100 bootstrap replicates. CRISPR/Cas9 target sites (protospacer with protospacer-adjacent motif “NGG”) in the genomic loci of interest were identified using the CRISPR Finder tool (default settings), with the *K. petricola* genome sequence as the off-target reference database. The protospacers used had at least four mismatches to the closest predicted off-target site. SnapGene 4.0.8 (GSL Biotech LLC) was used to design and document cloning strategies by generating sequence maps and aligning Sanger sequencing reads.

### Standard molecular methods

Genomic DNA was extracted as described by Voigt et al. (30). DNA was mixed with Midori Green Direct (Biozym Scientific) and separated in 1% agarose gels with the MassRuler DNA Ladder Mix (Thermo Scientific) or the 1 kb Plus DNA Ladder (New England Biolabs, NEB) as a size reference. PCRs were carried out with primers from Eurofins Genomics (Table S5), the Q5 High-Fidelity DNA polymerase (NEB) for cloning and sequencing, and the *Taq* DNA polymerase (NEB) for diagnostic PCR. Plasmids listed in Table S6 were assembled via homologous recombination in *S. cerevisiae* FY843 (101, 102), amplified in *Escherichia coli* DH5α (Invitrogen) and isolated from *S. cerevisiae* and *E. coli* with the Monarch Plasmid Miniprep Kit (NEB). Larger amounts of plasmid DNA were extracted from *E. coli* with the NucleoBond Xtra Midi Kit (Macherey-Nagel). PCR products were purified with the Monarch PCR and DNA Cleanup Kit (NEB) and sequenced with the Mix2Seq Kit at Eurofins Genomics. sgRNAs were synthesized *in vitro* with the EnGen sgRNA Synthesis Kit (NEB), purified with the Monarch RNA Cleanup Kit (NEB), and assembled in a molar ratio of 1:1 with EnGen Spy Cas9 NLS (NEB) by incubation for 10 min at 22°C.

### Genetic manipulation of *K. petricola*

The deletion of coding regions (knock-out, KO) and the targeted integration of expression constructs (knock-in, KI) into the *K. petricola* genome was accomplished by introducing double strand breaks (DSB) by the CRISPR/Cas9 technology and providing donor DNA for repair of the DSB by homologous recombination (HR) according to Voigt et al. (30) and Erdmann et al. (31). Target-specific ribonucleoproteins (RNP) were either expressed and assembled *in vivo* from plasmid DNA or added along with the donor DNA as pre-assembled RNP as specified in Table S7. Per approach, 1 × 10^6^ protoplasts were transformed with 10–15 µL of a PCR sample or a restriction digest (linear donor DNA) together with either 2 µg of sgRNA- and Cas9-delivering plasmid (circular DNA), or 1 µg of sgRNA assembled with 5 µg of Cas9, as described previously. The correct integration of deletion and expression constructs as a consequence of HR was detected by diagnostic PCR by combining primers binding upstream or downstream of the integration site with those binding within the integrated sequences, that is, in resistance or expression cassettes (Fig. S6, S8 and S10). For all genetic manipulation strategies, multiple independent transformants were generated and subjected to an initial phenotypic screening; representative data from one randomly selected transformant are presented.

### Determination of iron content in biomass

Solid –Fe, +Fe, and hFe medium covered with cellophane was inoculated with 50,000 cells. After 21 days, the biomass in the center of the Petri dish was taken off and dried at 65°C. A weighed amount was digested in polytetrafluoroethylene beakers with 1 mL of 9.8 M H_2_O_2_ and 1 mL of 14.3 M HNO_3_ at 150°C. After digestion, the acidic solution was evaporated, and the resulting pellet was dissolved in 4 mL of 1 M HNO_3_ via ultrasonication. The HNO_3_ solution containing the dissolved biomass residue was analyzed by inductively coupled plasma-optical emission spectroscopy (ICP-OES, Varian 720-ES) in the HELGES laboratory at GFZ, Potsdam (103) as described before (36) (see Method S1; Tables S8 and S9 for details). The analytical uncertainty used to interpret the sample results is quantified based on the accuracy and precision of the repeated measurement of quality control standards, the error of the analysis of the standards, and the contribution of the blank (i.e., the solution used to dilute the samples, see supplementary information). The iron content was quantified by dividing the amount of iron in the digest by the amount of biomass digested (µg g^−1^).

### Iron reduction assay

To obtain the ferric reduction potential of the biomass, the absorbance of the red BPS-Fe(II) complex was measured spectrophotometrically. Twenty milliliters of liquid –Fe medium in Erlenmeyer flasks was inoculated with 10^6^ CFU and placed on a shaker for 14 days in the dark at 25°C and 100 rpm. Afterward, the cultures were centrifuged, a sample of the supernatant was taken, and the pellet (biomass) was washed once with fresh –Fe medium. Fresh –Fe medium with 0.5 mM Fe(III)Cl_2_*6H_2_O and 0.5 mM BPS was added to the biomass and supernatant samples and incubated for 3 h in the dark at 25°C and 100 rpm. After another centrifugation step, the A_535_ was measured using a UV-vis spectrophotometer (Genesys 10S, Thermo Scientific). An abiotic control was used as a blank. The pellet was dried at 65°C for 1 day, and the dry weight was measured. The concentration of reduced iron was obtained from the measured A_535_ values by running the experiment with a standard series (0, 0.01, 0.025, 0.05, 0.1, 0.25, 0.5, and 1 mM Fe(II)SO_4_*7H_2_O) of reduced iron (Fig. S11a). Finally, the reduced iron concentration was divided by the dry weight and the duration of the incubation to obtain the reduction capacity in mM Fe(II) g^−1^ h^−1^. In the case of the supernatant samples, the reduced iron concentrations were divided by the dry weight of the respective culture, the fraction of supernatant analyzed, and the duration of the incubation.

### Statistical analyses

In general, all data are shown as the average of three or four independent replicates with two times the standard error (Table S10). Such a sample size is too low to test for normality. Differences between the strains and conditions were thus analyzed via the non-parametric Kruskal-Wallis test followed by the Conover-Iman post hoc test with Benjamini-Hochberg *P*-value adjustment (alpha level = 0.05). For both, the conover.test package (v1.1.6) in R software (v4.5.1; R core Team [104]) was used.

## Data Availability

The assembled genome of *K. petricola* A95 was deposited in GenBank under accession no. JBXBBG00000000. Nucleotide and protein sequences of studied *K. petricola* genes are available from GenBank; accession numbers are listed in Table S2. *K. petricola* strains generated in this study are available upon request.

## SUPPLEMENTAL MATERIAL

Supplemental material aem.02311-25-s0001.pdf Tables S2 to S9, Fig. S1 to S12, and Method S1.

Table S1 aem.02311-25-s0002.xlsx Iron acquisition-related proteins in Chaetothyriales.

Table S10 aem.02311-25-s0003.xlsx Raw data and statistical analysis results.

ASM does not own the copyrights to Supplemental Material that may be linked to, or accessed through, an article. The authors have granted ASM a non-exclusive, world-wide license to publish the Supplemental Material files. Please contact the corresponding author directly for reuse.
