# Supplementary material for: Iron acquisition strategies of the rock-inhabiting fungus Knufia petricola reveal vulnerability to chelator-based mitigation
Source: Appl Environ Microbiol. 2026 May 13;92(6):e02311-25. doi: 10.1128/aem.02311-25 (PMC13274355; doi:10.1128/aem.02311-25)
Supplement: Supplemental material — Tables S2 to S9, Fig. S1 to S12, and Method S1. [file aem.02311-25-s0001.pdf]

SUPPLEMENTARY MATERIAL

Iron acquisition strategies of the rock-inhabiting fungus *Knufia petricola* reveal vulnerability to chelator-based mitigation

Ruben Gerrits [1], Julia Schumacher [1,2], Anna A. Gorbushina [1,2]

[1] *Bundesanstalt für Materialforschung und -prüfung (BAM), Berlin, Germany*

[2] *Freie Universität Berlin, Berlin, Germany*

Table S1: Iron acquisition-related proteins in Chaetothyriales. (EXCEL file)

Table S2: *K. petricola* proteins putatively involved in iron acquisition and storage.

Table S3: *K. petricola* strains used in this study.

Table S4: Minimum inhibitory concentrations (MIC) of BPS and EDTA determined for other fungi.

Table S5: DNA oligonucleotides used in this study.

Table S6: Plasmids cloned in this study.

Table S7: Transformations of *K. petricola* protoplasts carried out in this study.

Table S8: Results of quality control standards analyzed by ICP-OES during analysis of samples.

Table S9: Concentrations of elements in the undiluted QC standard (with +Fe medium addition).

Table S10: Raw data and statistical analysis results. (EXCEL file)

Figure S1: *K. petricola* contains single copies of the two RIA components.

Figure S2: The putative ferric reductases (FREs) and NADPH oxidases (NOXs) of *K. petricola*.

Figure S3: The four putative siderophore transporters (SITs) of *K. petricola*.

Figure S4: Comparison of *K. petricola* NPS1 with SidC of ferricrocin-producing fungi.

Figure S5: The ferritin-like protein FER1 of *K. petricola*.

Figure S6: Deletion of *ftr1-fet1* and re-insertion of *ftr1-fet1* into Δ*ftr1-fet1* mutants.

Figure S7: Growth under low-iron and oxidative stress conditions are restored in Δ*ftr1-fet1*::*ftr1-fet1*.

Figure S8: Deletion and constitutive expression of *nps1* and the re-insertion of Δ*nps1* in Δ*nps1*.

Figure S9: Growth under iron-depleted conditions and siderophore secretion are restored in Δ*nps1*::*nps1*.

Figure S10: Deletion of *pks1* and complementation of the Δ*pks1* mutant.

Figure S11: The standard curve of the iron reduction assay and iron reduction capacities of supernatants.

Figure S12: Former panel shown as Figure 5e.

Supplementary Tables

Table S1: Iron acquisition-related proteins in Chaetothyriales. (EXCEL file)

Table S2: *K. petricola* proteins putatively involved in iron acquisition and storage.

Name	Protein description	Size	GenBank acc.	Putative function
**NPS1**	Nonribosomal peptide synthetase	4,897 aa	**WWL91118.1**	**Siderophore-mediated iron acquisition (SIA)**<br>Figure S3<br>Figure S4
**PPT1**	4'-phosphopantetheinyl transferase	366 aa	**WWL91116.1**	
**PPT2**	4'-phosphopantetheinyl transferase	262 aa	**WWL91117.1**	
**SIT1**	Siderophore iron transporter	600 aa	**WWL91119.1**	
**SIT2**	Siderophore iron transporter	597 aa	**WWL91120.1**	
**SIT3**	Siderophore iron transporter	613 aa	**WWL91121.1**	
**SIT4**	Siderophore iron transporter	578 aa	**WWL91122.1**	
**SIDJ**	Fusarinine C esterase	387 aa	**XHJ27574.1**	
**SIDL**	N^5-hydroxyornithine-acetylase	529 aa	**XHJ27575.1**	
**FRE1**	Ferric reductase	615 aa	**WWL91123.1**	**Reductive iron assimilation (RIA)**<br>Figure S1<br>Figure S2
**FRE2**	Ferric reductase	689 aa	**WWL91133.1**	
**FRE3**	Ferric reductase	701 aa	**WWL91124.1**	
**FRE4**	Ferric reductase	687 aa	**WWL91125.1**	
**FRE5**	Ferric reductase	544 aa	**WWL91126.1**	
**FRE6**	Ferric reductase	682 aa	**WWL91134.1**	
**FRE7**	Ferric reductase	863 aa	**WWL91127.1**	
**FRE8**	Ferric reductase	555 aa	**WWL91128.1**	
**FRE9**	Ferric reductase	551 aa	**XBA88317.1**	
**FRE10**	Ferric reductase	538 aa	**XBA88318.1**	
**FRE11**	Ferric reductase	551 aa	**XBA88319.1**	
**FRE12**	Ferric reductase	551 aa	**XBA88320.1**	
**FET1**	Multicopper oxidase/ferroxidase	628 aa	**WWL91131.1**	
**FTR1**	High-affinity iron permease	370 aa	**WWL91131.1**	
**FER1**	Ferritin-like protein	236 aa	**XHJ27572.1**	Internal iron storage
**SMF1**	Divalent metal ion transport	591 aa	**XHJ27576.1**	Low-affinity iron uptake
**MRS4**	Mitochondrial iron transporter	591 aa	**XHJ27577.1**	Mitochondrial iron import
**HMX1**	Heme oxygenase	331 aa	**XHJ27562.1**	Heme and siroheme metabolism
**HEM15**	Ferrochelatase	427 aa	**XHJ27563.1**	
**HEM14**	Protoporphyrinogen oxidase	713 aa	**XHJ27564.1**	
**HEM13**	Coproporphyrinogen III oxidase	445 aa	**XHJ27565.1**	
**HEM12**	Uroporphyrinogen decarboxylase	363 aa	**XHJ27566.1**	
**HEM4**	Uroporphyrinogen III synthase	386 aa	**XHJ27567.1**	
**HEM3**	Porphobilinogen deaminase	351 aa	**XHJ27568.1**	
**HEM2**	Aminolevulinate dehydratase	374 aa	**XHJ27569.1**	
**HEM1**	5-aminolevulinate synthase	609 aa	**XHJ27570.1**	
**HEM25**	Mitochondrial glycine transporter	331 aa	**XHJ27571.1**	
**MET1**	SAM uroporphyrinogen III transmethylase	599 aa	**XHJ27578.1**	
**MET8**	Bifunctional dehydrogenase/ferrochelatase	261 aa	**XHJ27579.1**	
**YFH1**	Mitochondrial matrix iron chaperone	263 aa	**XHJ27580.1**	
**SRE1**	GATA-type transcription factor	466 aa	**WWL91129.1**	Regulation of genes for iron acquisition
**SRB1**	SREB/bHLH transcription factor	913 aa	**WWL91130.1**	
**HAPX**	bZIP-type transcription factor	464 aa	**XHJ27573.1**	

Sequences were retrieved from genome assembly v1 of *K. petricola* A95 (unpublished) and submitted to GenBank.

Table S3: *K. petricola* strains used in this study.

Strain name	GMO ID	Genotype	Reference
**WT**	n/a	A95	C. Nai et al. (1)
**Δ*pks1***	KP-0033	A95; Δ*pks1* [(*nat1*::P*trpC*)]	O. Voigt et al. (2)
**Δ*pks1*::*pks1***	KP-0507	A95; Δ*pks1* [(*nat1*::P*trpC*)]; *igr2* [(T*niaD*::*nptII*::P*trpC*)–(P*pks1*::*pks1*::T*pks1*)]	This study
**Δ*ftr1-fet1***	KP-0133	A95; Δ*ftr1-fet1* [(*hph*::P*trpC*)]	This study
**Δ*ftr1-fet1*::*ftr1-fet1***	KP-0241	A95; Δ*ftr1-fet1* [(*hph*::P*trpC*)]; *igr2* [(T*niiA*::*nat1*::P*trpC*)–(T*ftr1*::*ftr1*::prom::*fet1*::T*fet1*)]	This study
**Δ*ftr1-fet1*/Δ*pks1***	KP-0261	A95; Δ*ftr1-fet1* [(*hph*::P*trpC*)]; Δ*pks1* [(T*niaD*::*nat1*::P*trpC*)]	This study
**Δ*nps1***	KP-0029	A95; Δ*nps1* [(*hph*::P*trpC*)]	This study
**Δ*nps1*::*nps1***	KP-0520	A95; Δ(*hph*::P*trpC*) [*nps1* [(*nps1*::T*gluc*)–(T*niiA*::*nptII*::P*trpC*)]	This study
**Δ*nps1*/Δ*ftr1-fet1***	KP-0088	A95; Δ*nps1* [(*hph*::P*trpC*)]; Δ*ftr1-fet1* [(T*niaD*::*nat1*::P*trpC*)]	This study
**Δ*nps1*/Δ*pks1***	KP-0263	A95; Δ*nps1* [(*hph*::P*trpC*)]; Δ*pks1* [(T*niaD*::*nat1*::P*trpC*)]	This study
**CE::*nps1***	KP-0271	A95; ΔP*nps1* [(T*niaD*::*hph*::P*trpC*)-(P*oliC*)]	This study

GMO ID – unique identifier assigned to genetically modified organisms in laboratory records.
CE – constitutive expression; Δ – deletion; in square brackets – recombinant sequences inserted into genome; in parentheses – resistance and expression cassettes with P*romoter*::*gene*::T*erminator*; *hph* – hygromycin B phosphotransferase from *E. coli* mediating resistance to hygromycin (HYG, H); *nat1* – nourseothricin N-acetyltransferase from *Streptomyces noursei* mediating resistance to nourseothricin (NTC, N); *nptII* – neomycin phosphotransferase II from *E. coli* mediating resistance to geneticin (G418, G); *igr2* – intergenic region 2 of *K. petricola* for neutral insertion of expression constructs; P*trpC,* P*oliC* – constitutive promoters from *Aspergillus nidulans*; T*niaD,* T*niiA* – terminators from *Botrytis cinerea.* For details on the editing strategies, see Figure S6 (*ftr1-fet1* strains), Figure S8 (*nps1* strains) and Figure S10 (*pks1*), and Table S7.

Table S4: Minimum inhibitory concentrations (MIC) of BPS and EDTA determined for other fungi.

Species	Lifestyle category	Pathogenic	Siderophore	MIC BPS (µM)	MIC EDTA (µM)	Method used to determine MIC	Reference for MIC
K. petricola	Rock inhabitant	No	Yes	150	250	Agar dilution method, CFU	(this study)
**Black fungus BAM-BF001**	Rock inhabitant	n/a	n/a	150	250	Agar dilution method, CFU	(this study)
**Black fungus BAM-BF027**	Rock inhabitant	n/a	n/a	150	200	Agar dilution method, CFU	(this study)
**Black fungus BAM-BF046**	Rock inhabitant	n/a	n/a	150	200	Agar dilution method, CFU	(this study)
Aspergillus fumigatus	Animal pathogen	Yes	Yes	> 400	n/a	Agar dilution method, drop	(3)
				n/a	> 8,200	Broth dilution method	(4)
Aspergillus niger	Animal pathogen	Yes	Yes	n/a	> 8,200	Broth dilution method	(4)
Saccharomyces cerevisiae	Epiphyte	No	No	500	750	Agar dilution method, CFU	(this study)
				n/a	55	Broth dilution method, IC_{50}	(5)
				n/a	1,000	Broth dilution method, 10^5 CFU ml^{-1} inoculant	(6)
				n/a	> 19,000	Broth dilution method, 10^7 CFU ml^{-1} inoculant	
Botrytis cinerea	Plant pathogen	Yes	Yes	n/a	2,510 *	Agar dilution method, drop, IC_{50}	(7)
Candida albicans	Animal pathogen	Yes	No	n/a	26,000	Broth dilution method	(8)
				3,000	n/a	Broth dilution method	(9)
Candida glabrata	Animal pathogen	Yes	No	n/a	3,300	Broth dilution method	(8)
Colletotrichum graminicola	Plant pathogen	Yes	Yes	1,000	n/a	Agar dilution method, CFU	(10)
Cryptococcus neoformans	Animal pathogen	Yes	No	n/a	220 *	Broth dilution method, IC_{50}	(5)
		Yes		n/a	250,000	Broth dilution method	(11)
Cryptococcus gattii	Animal pathogen	Yes	No	n/a	440 *	Broth dilution method, IC_{50}	(5)
Fusarium fujikuroi	Plant pathogen	Yes	Yes	n/a	1,500 *	Broth dilution method, IC_{90}	(12)
Fusarium oxysporum	Plant pathogen	Yes	Yes	n/a	> 8,200	Broth dilution method	(4)
Sporothrix schenckii	Animal pathogen	Yes	Yes	n/a	110 - 220 *	Broth dilution method, IC_{50}, filamentous form	(13)
				n/a	55 – 110 *	Broth dilution method, IC_{50}, yeast form	

The MIC values given are the concentrations at which there was a total inhibition of growth, unless stated otherwise: i.e., indicated with an * — IC_{50} and IC_{90} stand for an inhibition of 50 % and 90 %, respectively, of the initial response (growth). The agar dilution method was either applied by inoculating single cells (CFU) and counting the resulting colonies (agar dilution method, CFU) or by dropping an inoculant suspension with a known CFU titer and observing its growth (agar dilution method, drop assay). The growth with the broth dilution method is detected spectrophotometrically. Both methods are explained by Wiegand et al. (14).

Table S5: DNA oligonucleotides used in this study.

Name	Sequence (5'→ 3')	Features (5'→ 3')	Usage
***Kpftr1*-3U-5F**	gtaacgccagggttttcccagtcacga-GTGATGGCCGTGTAGGGACGG	pRS426 – *kpftr1*-3' non-coding	Cloning of pΔ*ftr1-fet1*-H [pEC0036] for amplification of replacement fragment with long-homologous (LH) sequences for deletion of *ftr1* and *fet1*
***Kpftr1*-3U-5R**	atccacttaacgttactgaaatctcca-CCCTGTCAGATGGCACGGTCG	pRS426 – *kpftr1*-3' non-coding	
***Kpfet1*-3U-3F**	ctccttcaatatcatcttctgtctccg-GTGTAAGCTGCTGGCTGCTTG	pRS426 – *kpfet1*-3' non-coding	
***Kpfet1*-3U-3R**	gcggataacaatttcacacaggaaaca-GTCCTTGGTCGGCAGATGAGGC	pRS426 – *kpfet1*-3' non-coding	
***Kpftr1*-SH5F**	agtcttttgttcaatcgaccgtgccatctgacagggctcatcgacaacttgccatgtcgc gcgtcgctgggcgcc-GCTAAGCGAGCGGGAGCTATCG	*kpftr1*-3' non-coding – T*niaD*	Amplification of replacement fragment with short-homologous (SH) sequences from pNDR-OGG for deletion of *ftr1* and *fet1*
***Kpfet1*-SH3R**	ccaacgtggttcaagcagccagcagcttacacttttggatcacgaagcatgccatgtcac gcccggtcaagtgat-GAATCGGGAATGCGGCTCCACAG	*kpfet1*-3' non-coding – P*oliC*	
***Kpfet1*-RNP-PS1**	ttctaatacgactcactata-g-gggatggtctattgataatc-gttttagagctaga	PT7 – g – *kpfet1* ^{PS1} – sgRNA	For *in-vitro* synthesis of sgRNA
***Kpftr1*-hi3R2**	GCATCTTGGCACGAGATTGACGCG	*kpftr1*-3' non-coding	Diagnostic PCRs for detection of deletion of *ftr1-fet1* or targeted integration of the *ftr1-fet1* complementation construct in *igr2*
***Kpfet1*-hi3R1**	CATCACCTCGGTGCCAATGCC	*kpfet1*-3' non-coding	
***Kpfet1*-wtF1**	GCTGGGAGACAGACCGTGCTCG	*kpfet1*-ORF	
***Kpfet1*-wtR1**	CTGAGGTTGCCGGATGTCATGAC	*kpfet1*-ORF	
***Kpfet1*-sF1**	CCGATACCGAAGTGAGGCCCGTG	*kpfet1*-3' non-coding	
***Kpftr1*-comF1**	aagcccaaaaaatgctccttcaatatc–GTGCTCGCTGGTGCTCAGTTAG	P*trpC* – *kpfrt1*-3'non-coding	Cloning of pIGR2N_*ftr1-fet* ^{COM} [pEC0081] for knock-in (KI) in *igr2*
***Kpfet1*-comR1**	ggtgttcccgaatggaattggaatacc-CTGGTGTGGCCTTCATGCAGCT	3R-adapter – *kpfet1*-3'non-coding	
***Kpnps1*-5F**	gtaacgccagggttttcccagtcacga-GACCGTCAGCCTTAGCAGCCGG	pRS426 – *kpnps1*-5' non-coding	Cloning of pΔ*nps1*-H [pEC0035] for amplification of replacement fragment with long-homologous (LH) sequences for deletion of *nps1* coding region
***Kpnps1*-5R**	atccacttaacgttactgaaatctcca-GGGAGGCTAGTCGGATCAGGC	pRS426 – *kpnps1*-5' non-coding	
***Kpnps1*-3F**	ctccttcaatatcatcttctgtctccg-GGGTTCGTGGAATTCCGTATGG	pRS426 – *kpnps1*-3' non-coding	
***Kpnps1*-3R**	gcggataacaatttcacacaggaaaca-CAATGACTAGCCGGTCGCAGCC	pRS426 – *kpnps1*-3' non-coding	
***Kpnps1*-RNP-PS1**	ttctaatacgactcactata-g-catatcctgggacggatctc-gttttagagctaga	PT7 – g – *kpnps1* ^{PS2} – sgRNA	For *in-vitro* synthesis of sgRNA
**T*niaD*-P*kpnps1*-SH5F**	tgccattaaagcaaactaattcggttgtaggatctacggctgcggtctggccaatttgga cgcacctatt-GCATTGGATTAATAATTGTTGCTAAGCGAG	P*kpnps1* – *B. cinerea* T*niaD*	Amplification of replacement fragment with SH sequences from pNDR-OGG for insertion of hygR-P*oliC* upstream of *nps1*
**P*oliC*-O*kpnps1*-SH3R**	ccggtaaagttcgccgattaggattggcaattgccaggcaaggctgtgtcgatggccgtt ccctggatgaagccat-GGTTGGATCGATTGTGATGTGATG	*kpnps1* ORF – *A. nidulans* P*oliC*	
***Kpnps1*-RNP-PS2**	ttctaatacgactcactata-g-agggtaaatcactcgggcta-gttttagagctaga	PT7 – g – *kpnps1* ^{PS1} – sgRNA	For *in-vitro* synthesis of sgRNA
**P*trpC*-sF2**	GCTTGGTGCACGATAACTTGGTG	*A. nidulans* P*trpC*	Cloning of pG-OCG [pEC0479] for COMiL constructs
***NptII*-T*niiA*-R**	tttgattccggttccagttccaatt-TCAGAAGAACTCGTCAAGAAGGCG	*B. cinerea* T*niiA* – *nptII*	
***Kpnps1*-pRS426-5F**	gtaacgccagggttttcccagtcacg-atttaaat-GAAGCGCTTCCTTACGGACCATTAC	pRS426-5F – *Swa*I – *kpnps1*-ORF	Cloning of pG-*nps1* ^{COMiL} [pEC0491] with partial *nps1*
***Kpnps1*-*Tgluc*-R**	taatcatacatcttatctacatacg-TCATCTATTACCGGCTGTAAGGCTTTC	*B. cinerea* Tgluc – *kpnps1*	

**Name**	**Sequence (5'→ 3')**	**Features (5'→ 3')**	**Usage**
***Kpnps1*-sR2**	GACGTGAGAATACAGCGACTCTTGC	*kpnps1* ORF	Amplification of fragments of *nps1* from *K. petricola* DNA or pG-*nps1*^{COMiL} *for in-vivo* assembly of a *nps1* complementation construct in the *K. petricola* Δ*nps1* [H] locus
***Kpnps1*-sF4**	CGATACCGCTCTGCACACTAGCAC	*kpnps1* ORF	
***Kpnps1*-comR1**	GCGTGGAGGATGTGCAACTCCAC	*kpnps1* ORF	
***Kpnps1*-comF2**	CGCGATACGAGCATGGTCAATGG	*kpnps1* ORF	
***Kpnps1*-sR3**	GACTGTCGAACAGCTCTTGGTCG	*kpnps1* ORF	
***Kpnps1*-sF3**	GAAGCGCTTCCTTACGGACCATTAC	*kpnps1* ORF	
**P*trpC*-SpeI-F1**	actagtGATATTGAAGGAGCATTTTTTGGGC	*Spe*I – *A. nidulans* P*trpC*	
***Kpnps1*-hi5F1**	GCCTCTTATCTGGTGCTGCATTGC	*kpnps1*-5' non-coding	Diagnostic PCRs for detection of deletion of *nps1* or targeted integration of hygR-P*oliC* or the complementation constructs *nps1*::T*gluc*-genR in-loco
***Kpnps1*-hi5F2**	GACGGCTTCATGCAGGTCAGCTG	*kpnps1*-5' non-coding	
***Kpnps1*-hi5F3**	CCTTAGCTGCGATTGACTTCTGGGC	*kpnps1*-5' non-coding	
***Kpnps1*-hiR**	GATGGCCGTTCCCTGGATGAAGCC	*kpnps1* ORF	
***Kpnps1*-hi3R**	GTGCGGATACACTTTGATACCCTG	*kpnps1*-3' non-coding	
***Kpnps1*-wtF1**	CCCAACGCCTCGACGTCTATG	*kpnps1* ORF	
***Kpnps1*-wtR1**	CTGTCCAAGCCATCAGGTCAGGTC	*kpnps1* ORF	
***Kpnps1*-sR1**	GAGCCTGAGGTATACATCACGTACG	*kpnps1* ORF	
***Kppks1*-RT5F**	gatcagcccttcttttgtttttctgctcgttaagaaccgcacccgaagtacgtcgacact cattcacatttcact-GCTAAGCGAGCGGGAGCTATCG	*kppks1*-5' (76 nt) – T*niaD*	Amplification of replacement fragment with SH sequences from pNDR-OGG for deletion of *pks1* coding region
***Kppks1*-RT3R**	gttgaaacaggttgcagtagttgatccagacaacaccatgatatgccagtcaagtgagta ggttcgtgtggttgc-GAATCGGGAATGCGGCTCCACAG	*kppks1*-3' (75 nt) – P*oliC*	
***Kppks1*-SH-hi5F**	GGTTGTCGGCAGTGATACGACAAG	*kppks1*-5' non-coding	Diagnostic PCR for detection of the replacement of the coding region of *pks1*
***Kppks1*-SH-hi3R**	GAGTTAGATTCGAGACACTCCACCAG	*kppks1*-3' non-coding	
***Kppks1*-wtF2**	GCCGATCTGGCATACACCACCAC	*kppks1* ORF	
***Kppks1*-wtR2**	GTCCGAGACGCCGTTGATGCATG	*kppks1* ORF	
***Kppks1*-P*trpC*-F**	gcccaaaaaatgctccttcaatatc-GTCTGGATGCTGCCCTATTAGAGTC	P*trpC* – *kppks1*-5' non-coding	Cloning of pIGR2G_*pks1*^{COM} [pEC0477] for targeted integration of a *pks1* complementation construct in *igr2*
***Kppks1*-com5R**	ACACCAGATACAGCCTCACCGTGACCAATGTTGGACTTGA	*kppks1* ORF	
***Kppks1*-wtF3**	GGCCGACTGAACTACTTCTTCAAG	*kppks1* ORF	
***Kppks1*-3RA-R**	gtgttcccgaatggaattggaatacc-CGAAGTTGCTTCTCGTACACATCG	3RA – *kppks1*-3' non-coding	
***Kpigr2*-RF-F1**	GCCTGCCAGAGTTCGGATTACCAG	*kpigr2*-5' non-coding	Amplification of constructs from pIGR2R-XXX for KI into *igr2*
***Kpigr2*-RF-R1**	GACCTGAGCTACAGGCCTCGATCG	*kpigr2*-5' non-coding	
***Kpigr2*-hi5F**	GAGCGATGTCGTCTCGGAAGATTAC	*kpigr2* non-coding	

Name	Sequence (5'→ 3')	Features (5'→ 3')	Usage
***Kpigr2*-hi3R**	GAGCAGCTAAGGTTGAGGAGCCC	*kpigr2* non-coding	Diagnostic PCRs for detection of targeted integration of expression constructs into *igr2*
***Kpigr2*-wtF1**	GGCAACTCAGACGCAATGGAGCC	*kpigr2* non-coding	
***Kpigr2*-wtR1**	CCTGAGCTACAGGCCTCGATCG	*kpigr2* non-coding	
***Hph*-F**	GTCGGAGACAGAAGATGATATTGAAGGAGC	*hph* ORF (hygR/H cassette)	Cloning of replacement fragments with LH and hygR
***Hph*-R**	GTTGGAGATTTCAGTAACGTTAAGTGGAT	*hph* ORF (hygR/H cassette)	
***Hph*-hiF**	GTCTGGACCGATGGCTGTGTAGAAG	*hph* ORF (hygR/H cassette)	Standard primers binding to modules in cloning vectors and replacement constructs used for colony PCR, sequencing and detection of HR events in *K. petricola* by diagnostic PCR
***Hph*-hiR**	GACAGACGTCGCGGTGAGTTCAG	*hph* ORF (hygR/H cassette)	
***Nat1*-hiF**	CGGCGAGCAGGCGCTCTACATGAGC	*nat1* ORF (natR/N cassette)	
***Nat1*-hiR**	GTACCGGTAAGCCGTGTCGTCGAG	*nat1* ORF (natR/N cassette)	
***NptII*-hiF**	GCCTTCTATCGCCTTCTTGACGAG	*nptII* ORF (genR/G cassette)	
***NptII*-hiR**	GCCGAATAGCCTCTCCACCCAAG	*nptII* ORF (genR/G cassette)	
**P*trpC*-P2**	CCTCCACTAGCTCCAGCCAAGCCC	*A. nidulans* P*trpC*	
**P*oliC*-sF2**	GGGAGACGTATTTAGGTGCTAGGG	*A. nidulans* P*oliC*	
**T*niiA*-hiF**	GTCATGCGTAGGGCACCGGTAGG	*B. cinerea* T*niiA*	
**3RA-hiF**	GTATTCCAATTCCATTCGGGAACACCAC	3R-adapter (3RA) in pIGRXR	

Lowercase letters – 5' overhangs for mediating homologous recombination in *S. cerevisiae* or *K. petricola*, or Gibson DNA assembly, or additional sequences; uppercase letters – 3' sequences annealing to the template DNA.
Goi-RNP-PS1 oligonucleotides are designed for sgRNA synthesis using the EnGen sgRNA Synthesis Kit (NEB).

Table S6: Plasmids cloned in this study.

**Name** [ID] (size)	**Entry plasmid**	**Amplicon 1**	**Amplicon 2**	**Amplicon 3**
**pΔ*ftr1-fet1*-H** **[pEC0036]** (8.771kb)	**pRS426** (15) digested w/ *Eco*RI + *Xho*I	**P*trpC*::*hph*** (1.443 kb) primers: *hph*-F/*hph*-R template: pCSN44 (16)	***kpfrt1* 5' LH flank** (1.158 kb) primers: *kpfrt1*-3U-5F/*kpfrt1*-3U-5R template: *K. petricola* DNA	***kpfet1* 3' LH flank** (0.806 kb) primers: *kpfet1*-3U-3F/*kpfet1*-3U-3R template: *K. petricola* DNA
**pIGR2N_*ftr1-fet* COM** **[pEC0081]** (17.285 kb)	**pIGR2N v1** [pEC0063] (17) digested w/ *Pac*I	**T*kpfet1*–T*kpftr1*** (5.616 kb) primers: *kpftr1*-comF1/*kpfet1*-comR1 template: *K. petricola* DNA	n/a	n/a
**pΔ*nps1*-H** **[pEC0035]** (9.851 kb)	**pRS426** (15) digested w/ *Eco*RI + *Xho*I	**P*trpC*::*hph*** (1.443 kb) primers: *hph*-F/*hph*-R template: pCSN44 (16)	***kpnps1* 5' LH flank** (1.797 kb) primers: *kpnps1*-5F/*kpnps1*-5R template: *K. petricola* DNA	***kpnps1* 3' LH flank** (1.109 kb) primers: *kpnps1*-3F/*kpnps1*-3R template: *K. petricola* DNA
**pG-OCG** **[pEC0479]** (9.383 kb)	**pN-OCG** [pEC0460] (18) digested w/ *Kpn*I + *Sph*I	***nptII* ΔNcoI** (0.951 kb) primers: P*trpC*-sF2/*nptII*-T*niiA*-R template: pNDG-OGG v2 [pEC0270] (17)	n/a	n/a
**pG-*nps1* COMiL** **[pEC0491]** (11.948 kb)	**pG-OCG** [pEC0479] (this study) digested w/ *Sac*II + *Not*I	***nps1* partial** (4.188 kb) primers: *kpnps1*-pRS426-5F/*kpnps1*-T*gluc*-R template: *K. petricola* DNA	n/a	n/a
**pIGR2G-*pks1* COM** **[pEC0477]** (21.076 kb)	**pIGR2G** [pEC0291] (17) digested w/ *Pac*I	**P*kppks1*::*pks1* [1]** (6.543 kb) primers: *kppks1*-P*trpC*-F/*kppks1*-com5R template: *K. petricola* DNA	***kppks1*::T*kppks1* [2]** (5.479 kb) primers: *kppks1*-wtF3/*kppks1*-3RA-R template: *K. petricola* DNA	n/a

Competent *S. cerevisiae* FY834 cells were transformed with 150 ng of the digested entry plasmid and 5 µl of each amplicon (insert DNA). Plasmid DNA from uracil-prototrophic cells was extracted and introduced into *E. coli* DH5α. Ampicillin-resistant *E. coli* colonies were screened for correctly assembled plasmids by PCR. Plasmid DNA from positive clones were extracted and sequenced with appropriate primers to confirm the error-free assembly.

Table S7: Transformations of *K. petricola* protoplasts carried out in this study.

Strain	GMO ID	#	Recipient	Donor DNA	CRISPR/Cas9
**Δ*pks1*::*pks1***<br>[N/G]	KP-0507	T1, 4, 5, 6	**Δ*pks1***<br>[KP-0033]<br>**PN2**	**[(T*niaD*::*nptII*::P*trpC*)–(P*kppks1*::*kppks1*::T*kppks1*)]** $^{\text{kpigr2-LH}}$ (15.534 kb)<br>isolated by digestion with *Swa*I<br>from pIGR2G-*pks1* ^{COM} [pEC0477] (this study)	**pAMA/ribo-*kpigr2*** $^{\text{PS1}}$ [pEC0059] (17) (transient expression, *in-vivo* assembly)
**Δ*ftr1-fet1***<br>[H]	KP-0133	T4.2, 4.3, 4.4	**WT:A95**	**[(*hph*::P*trpC*)]** $^{\Delta\text{kpftr1-fet1-LH}}$ (3.323 kb)<br>primers: *kpftr1*-3U-5F/*kpfet1*-3U-3R<br>template: pΔ*ftr1-fet1*-H [pEC0036] (this study)	**RNP-*kpfet1*** $^{\text{PS1}}$<br>(*in-vitro* synthesized and assembled)
**Δ*ftr1-fet1*::*ftr1-fet1***<br>[H/N]	KP-0241	T1, 2, 3, 4	**Δ*ftr1-fet1***<br>[KP-0133]<br>**T4.2**	**[(T*niiA*::*nat1*::P*trpC*)–(T*kpftr1*::*kpftr1*::prom::*kpfet1*::T*kpfet1*)]** $^{\text{kpigr2-LH}}$ (8.651 kb)<br>primers: *kpigr2*-RF-F1/*kpigr2*-RF-R1<br>template: pIGR2N_*ftr1-fet* ^{COM} [pEC0081] (this study)	**pAMA/ribo-*kpigr2*** $^{\text{PS1}}$ [pEC0059] (17) (transient expression, *in-vivo* assembly)
**Δ*ftr1-fet1*/Δ*pks1***<br>[H/N]	KP-0261	T1, 2, 3	**Δ*ftr1-fet1***<br>[KP-0133]<br>**T4.2**	**[(T*niaD*::*nat1*::P*trpC*)]** $^{\Delta\text{kppks1-SH}}$ (1.473 kb)<br>primers: *kppks1*-RT5F/*kppks1*-RT3R<br>template: pNDN-OGG (19)	**pAMA/ribo-*kppks1*** $^{\text{PS2}}$ [pEC0028] (2) (transient expression, *in-vivo* assembly)
**Δ*nps1***<br>[H]	KP-0029	T1.1, 1.2, 2.2	**WT:A95**	**[(*hph*::P*trpC*)]** $^{\Delta\text{kpnps1-LH}}$ (4.403 kb)<br>primers: *kpnps1*-5F/*kpnps1*-3R<br>template: pΔ*nps1*-H [pEC0035] (this study)	n/a
**Δ*nps1*::*nps1***<br>[G]	KP-0520	T1, 2, 3	**Δ*nps1***<br>[KP-0029]<br>**T1.1**	***kpnps1*-C1** (4.820 kb); primers: *kpnps1*-hi5F/*kpnps1*-sR2; template: *K. petricola* DNA<br>***kpnps1*-C2** (4.653 kb); primers: *kpnps1*-sF4/*kpnps1*-comR1; template: *K. petricola* DNA<br>***kpnps1*-C3** (4.519 kb); primers: *kpnps1*-comF2/*kpnps1*-sR3; template: *K. petricola* DNA<br>***kpnps1*-C4** (6.431 kb); primers: *kpnps1*-sF3/P*trpC*-SpeI-F1; template: pG-*nps1* ^{COMiL} [pEC0491]	**pAMA/tRNA-*hph*** $^{\text{PS1}}$ [pEC0461] (18) (transient expression, *in-vivo* assembly)
**Δ*nps1*/Δ*ftr1-fet1***<br>[H/N]	KP-0088	T1, 2, 3	**Δ*nps1***<br>[KP-0029]<br>**T1.1**	**[(T*niaD*::*nat1*::P*trpC*)]** $^{\Delta\text{kpftr1-fet1-SH}}$ (1.473 kb)<br>primers: *kpftr1*-SH5F/*kpfet1*-SH3R<br>template: pNDN-OGG (19)	**RNP-*kpfet1*** $^{\text{PS1}}$<br>(*in-vitro* synthesized and assembled)
**Δ*nps1*/Δ*pks1***<br>[H/N]	KP-0263	T1, 2, 3	**Δ*nps1***<br>[KP-0029]<br>**T1.1**	**[(T*niaD*::*nat1*::P*trpC*)]** $^{\Delta\text{kppks1-SH}}$ (1.473 kb)<br>primers: *kppks1*-RT5F/*kppks1*-RT3R<br>template: pNDN-OGG (19)	**pAMA/ribo-*kppks1*** $^{\text{PS2}}$ [pEC0028] (2) (transient expression, *in-vivo* assembly)
**CE::*nps1***<br>[H]	KP-0271	T1, 2, 3, 4	**WT:A95**	**[(T*niaD*::*hph*::P*trpC*)–(P*oliC*)]** $^{\text{Pkpnps1-SH}}$ (2.697 kb)<br>primers: T*niaD*-P*kpnps1*-SH5F/P*oliC*-O*kpnps1*-SH3R<br>template: pNDH-OGG (19)	**RNP-*kpnps1*** $^{\text{PS2}}$<br>(*in-vitro* synthesized and assembled)

For details on the editing strategies see, Figure S6 (*ftr1-fet1* strains), Figure S8 (*nps1* strains) and Figure S10 (*pks1*). For constructs cloned in this study, see Table S6.
GMO ID – unique identifier assigned to genetically modified organisms in laboratory records; # – number designating individual transformants; CE – constitutive expression. RNP – ribonucleoprotein (Cas9 with sgRNA).

Table S8: Results of quality control standards analyzed by ICP-OES during analysis of samples.

**Name**	**Fe (µg g^{-1})**	**In (µg g^{-1})**	**Sc (µg g^{-1})**
QC Standard 1: (Merck CertiPUR ICP standards + MM+Fe medium solution without agar) reference value	5.48	1.98	1.96
Measured value (n=7)	5.43	1.71	1.95
2SD	0.16	0.03	0.02
Measured deviation from reference value (%)	-0.9 %	-13.5 %	-0.6 %
QC Standard 2: (Merck CertiPUR ICP standards + H_2O) reference value	3.92	1.98	1.96
Measured value (n=7)	4.00	2.01	2.03
2SD	0.08	0.05	0.04
Measured deviation from reference value (%)	2.2 %	1.7 %	3.8 %

All analyses were performed in the Helmholtz Laboratory for the Geochemistry of the Earth Surface (HELGES) at GFZ Potsdam. A QC standard stock solution was prepared in dilute HNO_3. QC standard 1 contained the MM+Fe medium components (at the same final concentrations as used in the Fe uptake experiment, Figure 5c) mixed with several ICP single-element standard solutions (Merck CertiPUR). Before analysis, all standard stock solutions were diluted twice with 0.29 M HNO_3 containing ca. 2000 ppm Cs, ca. 2 ppm In and ca. 2 ppm Sc. Note that concentration values are corrected for dilution. The undiluted concentrations of the QC standards prepared from stock before each analytical session are given in Table S9. The mean results of seven replicates measurements are given together with two standard deviations (2SD), a quantitative indication of the precision. The measured deviation from the reference value is a quantitative indication of accuracy.

Table S9: Concentrations of elements in the undiluted QC standard (with +Fe medium addition).

Element	Concentration (µg g^{-1}) in QC standard 1	Concentration (µg g^{-1}) in QC standard 2
Al	3.75	3.83
C	8767.40	0.00
Ca	7.64	3.94
Cr	3.86	3.94
Cu	3.91	3.93
Fe	5.48	3.92
In	0.00	0.00
K	464.10	0.00
Mg	50.34	3.94
Mn	3.91	3.94
Na	764.98	0.00
Ni	3.86	3.94
P	171.80	0.00
S	433.87	0.00
Sc	0.00	0.00
Si	3.86	3.95
Sr	3.81	3.90
Ti	3.87	3.95
Cs	0.00	0.00
HNO_3 (mol l^{-1})	0.15	0.15

Table S10: Raw data and statistical analysis results. (EXCEL file)

Supplementary Methods

Method S1: Inductively coupled plasma optical emission spectroscopy: sample preparation, analysis protocol, quality control standards and medium blanks

To determine the elemental composition of the biomass samples, they were analyzed by inductively coupled plasma optical emission spectroscopy (ICP-OES, Varian 720-ES). Sample preparation and analyses were performed in the Helmholtz Laboratory for the Geochemistry of the Earth Surface (HELGES) at GFZ Potsdam (20). The analytical procedure and instrumentation are described in J. A. Schuessler et al. (21) and was followed with minor adaptations. First, weighed and dried biomass samples were digested in PTFE beakers with 1 ml of 9.8 M H_2O_2 and 1 ml of 14.3 M HNO_3 at 150 °C. After digestion, the acidic solution was evaporated, the resulting pellet was dissolved in 4 ml of 1 M HNO_3 via ultrasonication and stored in acid-cleaned, polypropylene falcon tubes at 4 °C. At the day of analysis, samples were diluted 1:2 with quartz-distilled HNO_3 containing Cs as ionization buffer to achieve matrix matching to the calibration standards. In and Sc were added as internal recovery standards. The final concentrations in diluted sample solutions were 0.29 M HNO_3, 1000 ppm Cs, 1.0 ppm In and 1.0 ppm Sc, matching the composition of the ICP-OES calibration standards. Linear calibration range was verified by measurements of 4 to 8 calibration standard solutions, covering the range of element concentrations in the diluted samples. Calibration standards were prepared from multi-element ICP standards (Merck CertiPUR, traceable to NIST reference materials) diluted in 0.3 M HNO_3 containing ca. 1 mg g^{-1} Cs prepared from $CsNO_3$ salt (99.999 % purity). Uncertainties from calibration were typically below 3 % relative. The detection limit for Fe was 0.0637 ± 0.0009 µg g^{-1}. Quality control standards (QCs) (Multi-element ICP-MS standard mixed with the medium solution or H_2O) were analyzed after every 10th sample. The analytical uncertainty used to interpret the sample results is conservatively estimated based on the accuracy and the precision of the repeated measurement of the QCs. Analytical results of these QCs are reported in Table S8 and their element concentrations are given in Table S9. To elucidate the element contributions from the HNO_3 + Cs + In + Sc solution to the measured Mg concentrations in the samples, blanks were analyzed. The Fe concentration values of the blank (i.e. 0.0091 ± 0.0001 ppm) were subtracted from the Fe concentrations of the biomass samples.

Supplementary Figures

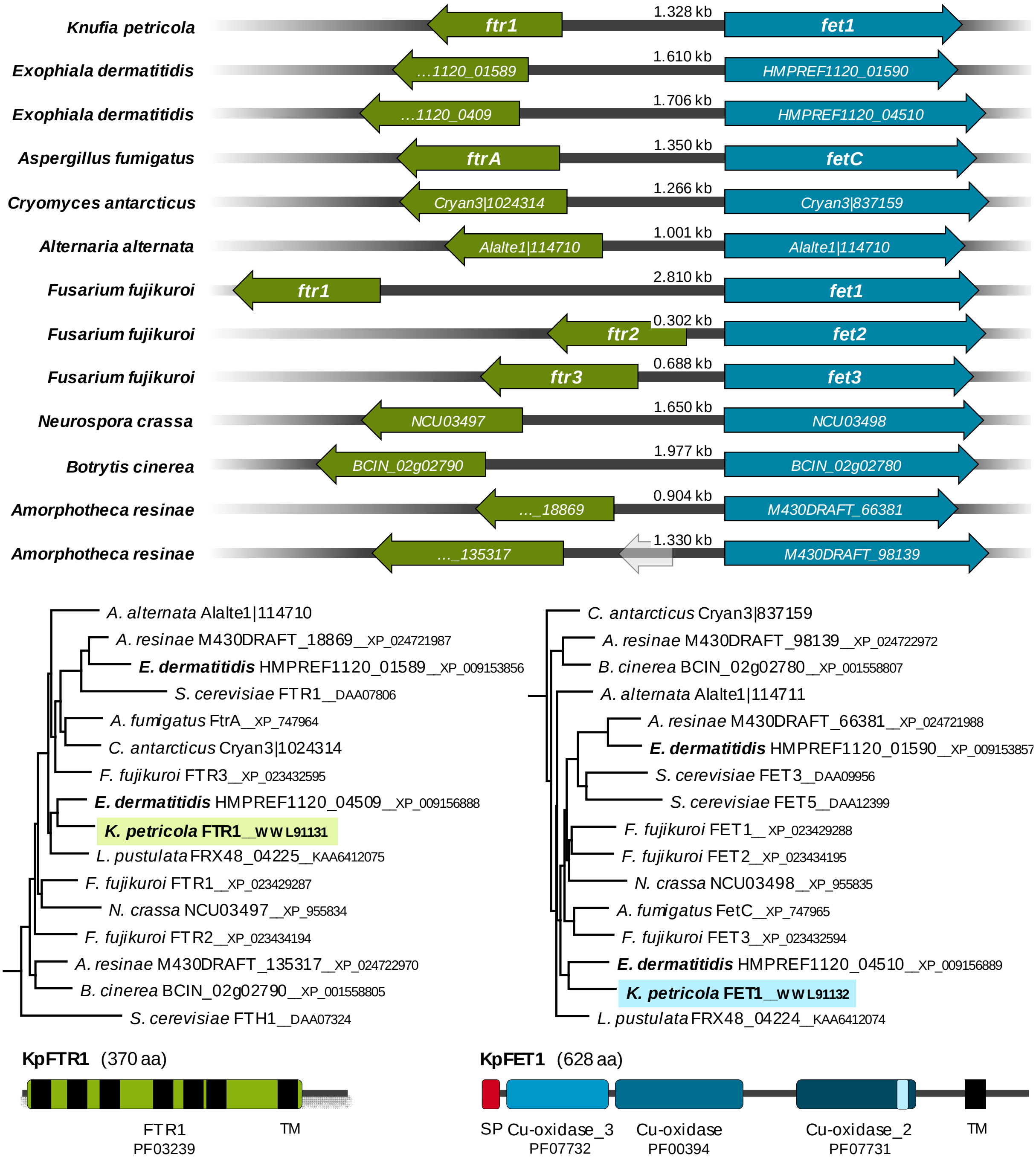

Figure S1: ***K. petricola* contains single copies of the two RIA components.**
Genes encoding the iron permease (FTR) and the ferroxidase (FET) are often physically linked. Genes are not linked in the genome of *S. cerevisiae* (not shown). The genes are separated by bidirectional promoter regions of 0.3 to 2.8 kb in the Ascomycetes belonging to different classes: Eurotiomycetes/Eurotiales (*A. fumigatus*), Eurotiomycetes/Chaetothyriales (*K. petricola*, *E. dermatitidis*), Lecanoromycetes (*Lasallia pustulata*), Dothideomycetes (*A. alternaria*, *C. antarcticus*), Sordariomycetes (*F. fujikuroi*, *N. crassa*), Leotiomycetes (*B. cinerea*, *A. resinae*). *K. petricola* FTR1 and FET1 group with one FTR/FET out of two copies of *E. dermatitidis* in a phylogenetic tree. The *K. petricola* proteins with the characteristic domains are shown. TM – transmembrane domain, SP – signal peptide for secretory pathway.

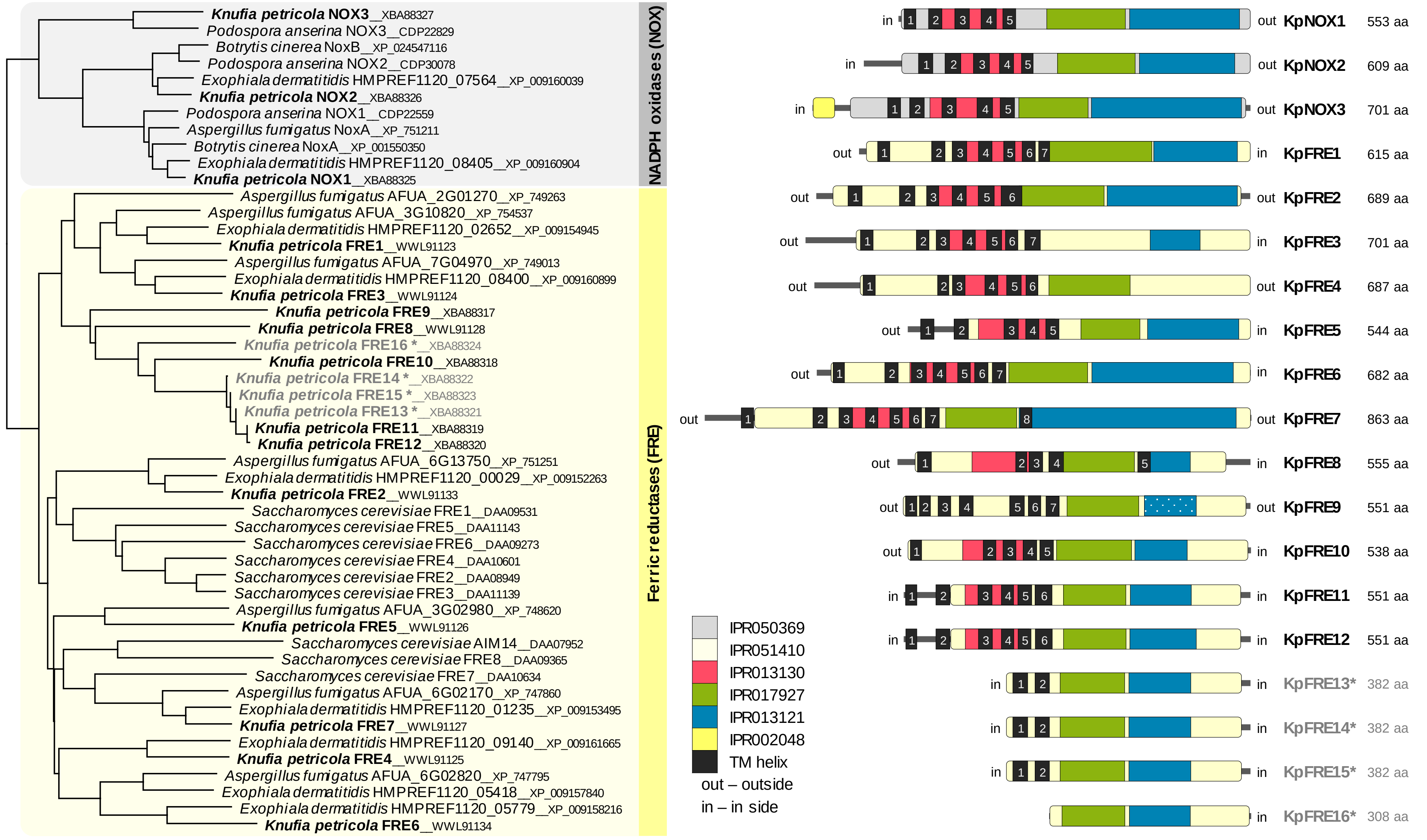

Figure S2: The putative ferric reductases (FREs) and NADPH oxidases (NOXs) of *K. petricola*.
Asterisks indicate truncated *K. petricola* proteins, which are unlikely functional FREs. IPR050369 – Respiratory burst oxidase/Ferric reductase, IPR051410 – Ferric/Cupric Reductase Transmembrane Component, IPR013130 – Ferric reductase transmembrane component-like domain, IPR017927 – FAD-binding domain, ferredoxin reductase-type, IPR013121 – Ferric reductase, NAD binding domain, IPR002048 – EF-hand domain. TM – transmembrane. The predicted localization of the N/C termini (inside/outside of cell) are indicated.

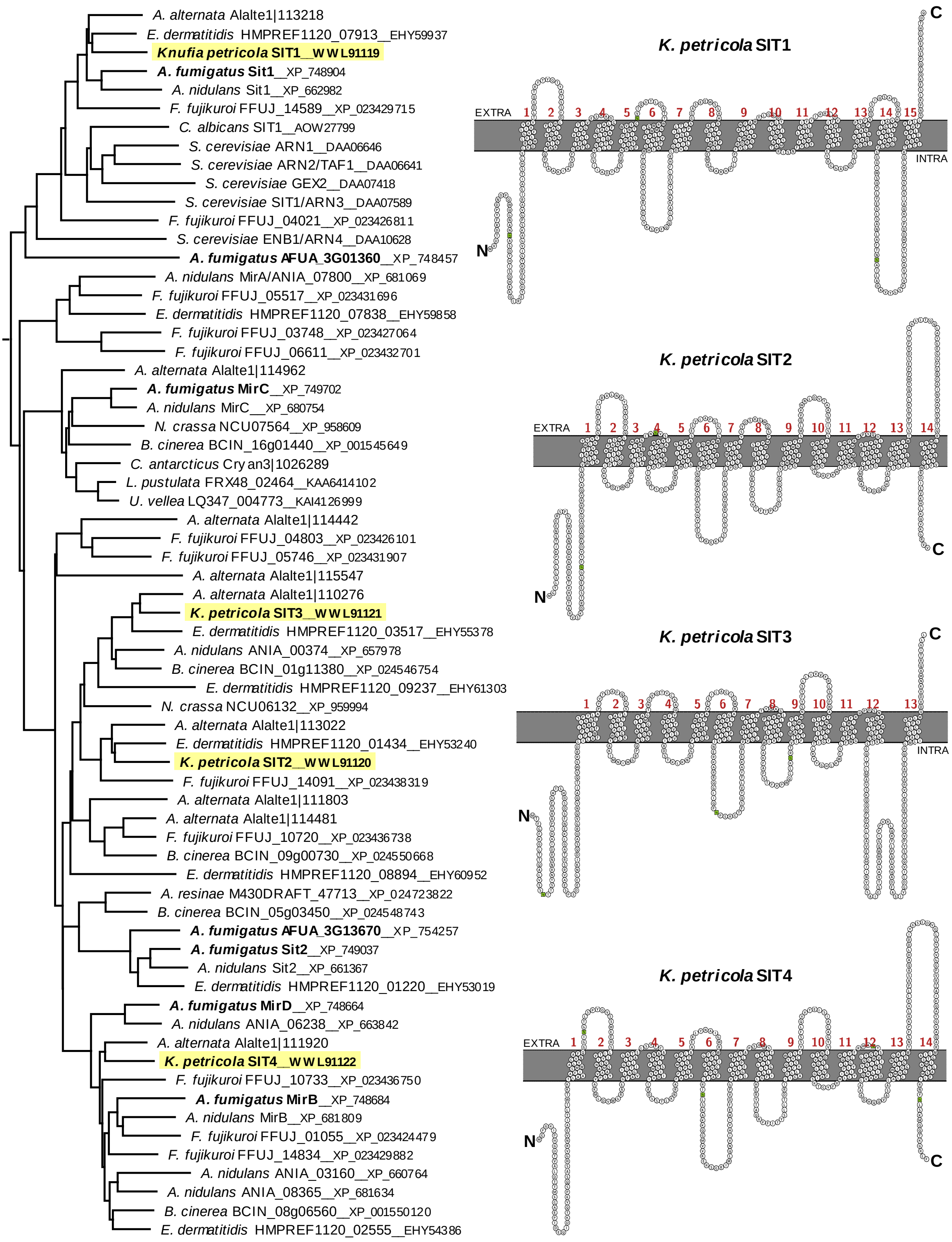

Figure S3: The four putative siderophore transporters (SITs) of *K. petricola*.
The *K. petricola* proteins are highlighted. The transmembrane regions were visualized with PROTTER v1.0 (22)

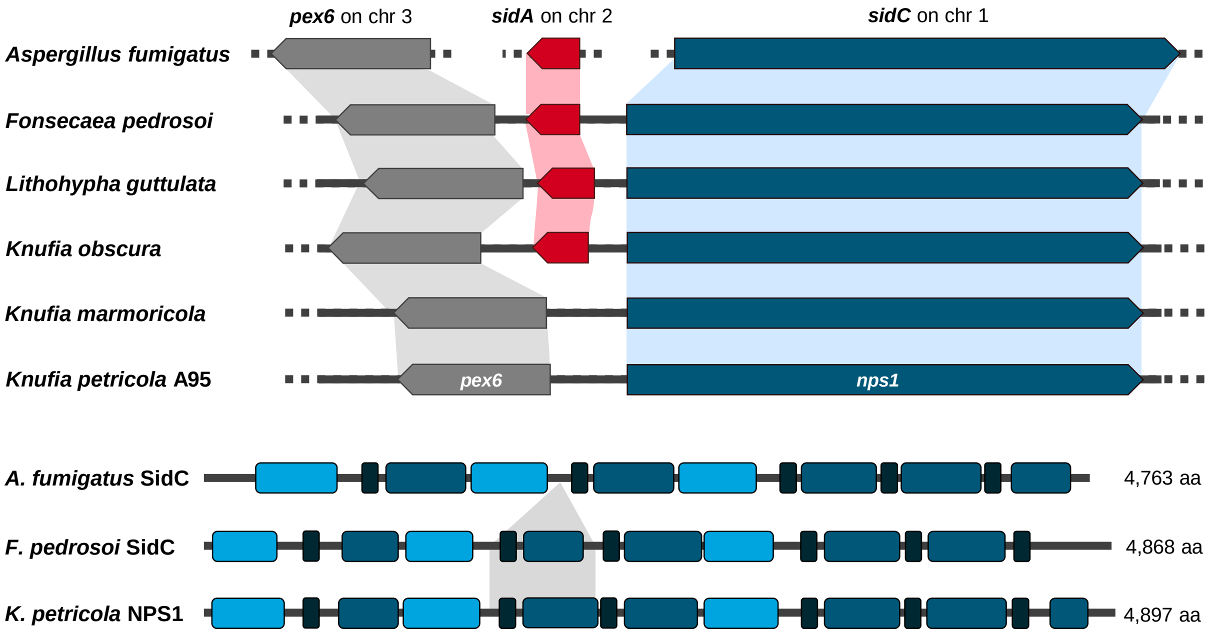

Figure S4: Comparison of *K. petricola* NPS1 with SidC of ferricrocin-producing fungi.
Above: Genomic organization of *nps1* and *sidA-sidC* in different Eurotiomycetes. *pex6* encodes a peroxin involved in the biogenesis of peroxisomes. Below: The domain structure of NPS1 resembles those of the verified ferrocrocin-producer *F. pedrosoi*. Conserved domains of NRPSs are shown as boxes: light – Adenylation/AMP-dependent enzyme domain [PF00501], dark blue – Condensation domain forming the amide bond [PF00668], black – Thiolation domain/phosphopantetheine attachment site [PF00550]. For GenBank accessions see Table S1.

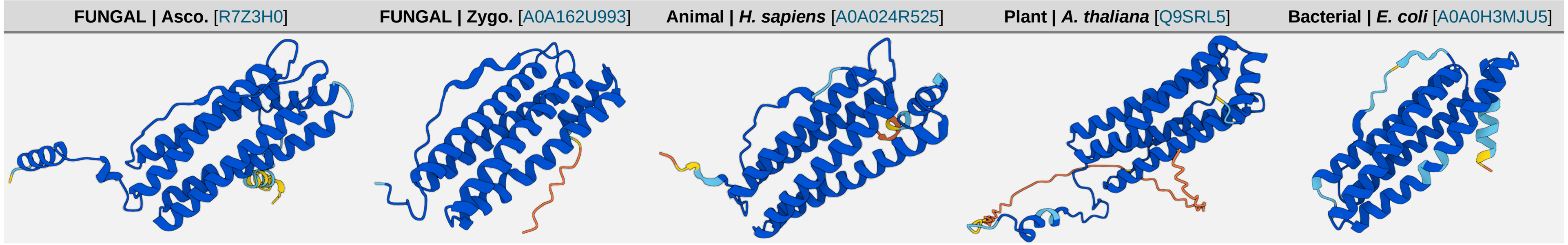


```
                                     helix A                    helix B
K. petricola      aa 1   MSASGEVSG-NTEQVQKALKEHTK------NNIDEYIRVSTFSDEKVDDAIRGHISLEWKTWFFYRKLGADCGRANVGLHGFAMLFKRAAAECFADGMWLESYLVQRGGRSKPSDIPAPDVEF-PDDPVDPV
K. marmoricola    aa 1   MSASGEVSG-NTEAVQKALKEHTK------NNIDEYVQVSTFSDEEVHESIRGHISLEWKTWFMYRKLGADCGRANIGLHGFAMLFKRAAAECFADGMWLESYLVQRGGRSKPSDIPAPDIHF-PDDPVDPV
L. guttulata      aa 1   MSSGDQQSGKDTQQVQQALREHTE------ANIDDYVKTSTFTDNDLHESIRGHIALEWKTWFFYRKMGADCGRANVGLHGFAQLFKRAAAECFADGMWLESYLIQRGGRSNPSDIPAPNTEF-PDNPVDPV
C. apollinis      aa 1   MS--------HTEEVQQAIEKHSK------ANIDEYIQASSFKPD-LQDSIRGHIALNWKSWFFYRKLSADCARANVGLHGFSMLWKRAATECFADGMWLEGYLVQRGGRSKPSDIPAPNVQW-PDDPVDPV
H. werneckii      aa 1   MASEGDPVKKHTGFVQEQLKAHSK------NNIDEYIQTASFDET-VENAVRGHIHIEWQSWFFYRKLAADAARANIGLHGFAMLFKRAAAECFADGMWLENYLVQRGGRSAPSDIPAPKVHF-PDNPIDPV
F. oxysporum      aa 1   MS------GSNTDKVQENLKKFSA------ENIDAHIQASTFTDD-IQNAIRQHIQTEYKAWFFFRKLGADCLRSNVSLHGFAALWKRSAQEAFADATWLESYLVQRGGRAKPSDIPAPDIEW-PDDPVDPV
A. fumigatus      aa 1   MS----------EQARAIISEISG------HDLDQWLRPSTFTEE-LEESIRGHIHEELTSWMFYRKLAADCSRANIALHGFAMLWHRSAKECLIDMEWLEKYLIIRGGRCKPTNIEAPKYEW-PDSPVEPV
P. blakesleeanus  aa 1   MS----------------------------------LAKQTFATT-SEEAINQQINTELQASQVYLSMASWAQHSSVALPGLEKYFREQAEEERKHAQHLIDYQNTRGGKVILRSLQAP-----ETEWKSAK
R. microsporus    aa 1   M----------------------------------ALTKQTFSTQ-SEEALNQQINTELQASQVYLSMSAWAQHSTVALPGLEKYFREAAEEEREHAQKLIDYVNTRGGRVALRALHAP-----EVDWKSAK
O. edulis         aa 1   MS--------------------------------QSQPRQTFHEE-SEAGINRQINMELYASYTYQSMAFYFDRDDVALPGFAKFFRDSSSEEREHAEKLMKYQNKRGGRIVLQDIKKPD----RDEWGTGL
H. sapiens        aa 1   M-----------------------------TTASTSQVRQNYHQD-SEAAINRQINLELYASYVYLSMSYYFDRDDVALKNFAKYFLHQSHEEREHAEKLMKLQNQRGGRIFLQDIKKPD----CDDWESGL
A. thaliana       aa 46  ..AKGTNTKSLTGVVFEPFEEVKKEMELVPTTPFVSLARHKFSDD-SESAINDQINVEYNVSYVYHALYAYFDRDNVGLKGFAKFFNDSSLEERGHAEMFMEYQNKRGGRVKLQSILMPVSEFDHEEKGDAL
Z. mays           aa 44  ..AAGKGKEVLSGVVFQPFEEIKGELALVPQSPDRSLARHKFVDD-CEAAINEQINVEYNASYAYHSLFAYFDRDNVALKGFAKFFKESSDEEREHAEKLMEYQNKRGGRVRLQSIVAPLTEFDHPEKGDAL

                         helix C                helix D
K. petricola      IPVHAALMREKELLEDLLRLCKAADGAGDYALEDAIETRFLQKETKHVKDLGDLLQQCVRIS-KNVGHGLYHLDKELRETKGLVPWANNNNPDSTDDILMSAMKDLSEAKLFP----------------*  236 aa
K. marmoricola    IPVHAAFQREKELLEDLLRLCKTADEAGDYALEDAIETRFLKKETKHVKDMGDLLQQCVRIS-KNVGHGLYHLDKELRESKGIVPWAHNNNPDSTDEILSAAMGDLAEAKLFP----------------*  236 aa
L. guttulata      RPVKEALDHEKALLEDLLRLCKMADSKEDYALEDVLETRFLKKETKHVKDLGDLLQQCVRIS-KQVGHGLYHLDKELRENSGVIPWSRNNNPDYTDELLAAAMKDLGDTKLFPN---------------*  238 aa
C. apollinis      QPVYEALQVEKELLEDVLRLCKAADEAEDYALEDVLETRFLKKETKHVKDLGDLLQQCVRIS-KQPGHGLYHLDKELRETKGVVPWSQANNPDNTDLLLQAATKDLSNTALFPS---------------*  229 aa
H. werneckii      IPAHEALQAEKALLEDLLRLCKAADQAGDYALEDVIETRFLQKESKHVKDLGDLLQQCVRVS-KDVGHGVYHLDKELRSTKGLVPWHVANDPDTTDRLLEETMTDLRETKLF-----------------*  235 aa
F. oxysporum      QPVYAALQTEKEILEDLHRLCAAAEKAGDNALEDVIESRFLRKETRHVKDMGDLLQQCVRIS-KQAGHGLYHLDKELRANNGVVPWASFNDPDKSDELLRGVVKDLYKAAV------------------*  228 aa
A. fumigatus      GPCREAFNVQRNLFVDLEKLCTLAEKCRDTAIVNAIESRFLRKHAKHLKNLGDLLQQVTRVS-KQPGLGLYHLDAELRHNNGCIPWMSMNDPDTIEEGIRAVTKKIGGGLALQTHHEGTKLASKEVGHC*  242 aa
P. blakesleeanus  NAIESALQLEKDVNKSLLNLHKIGDSNGDPQMCDFVEATYLGEQVEAIKTLADMVTQLNRVG---EGLGVYLWDQQLYREGTGAGSRAISRAD------------------------------------*  182 aa
R. microsporus    NAVESALQLEKDVNHSLLKLHKIADSNSDPQMCDFIEGNFLGEQVEAIKKLSDMVTQLNRVG---EGLGLYLWDQQLYRDGTGAGSRAVGTPHHG----------------------------------*  184 aa
O. edulis         DAMQMALQLEKTVNQSLLDLHKVAGSHNDAQMCDFIESEYLEEQVQAIKEVSDHITQQLKRVG--TGLGEYMYDKQLQS--------------------------------------------------*  171 aa
H. sapiens        NAMECALHLEKNVNQSLLELHKLATDKNDPHLCDFIETHYLNEQVKAIKELGDHVTNLRKMGAPESGLAEYLFDKHTLGDSDNES--------------------------------------------*  183 aa
A. thaliana       HAMELALSLEKLTNEKLLKLQSVGVKNNDVQLVDFVESEFLGEQVEAIKKISEYVAQLRRIG---KGHGVWHFDQMLLNDEV-----------------------------------------------*  253 aa
Z. mays           YAMELTLALEKLVNEKLHSLHGVATRCNDPQLIDFIESEFLEEQVEAINKVSKYVAQLRRVG--NKGHGVWHFDQMLLQEGA-----------------------------------------------*  252 aa
```

Figure S5: The ferritin-like protein FER1 of *K. petricola*.
Predicted structures of different ferritins from the AlphaFold Protein Structure Database (https://alphafold.ebi.ac.uk/). Sequence alignment of ferritins from: Ascomycota – *K. petricola* [XHJ27572.1], *K. marmoricola* [KAK5092228.1], *L. guttulata* [KAK6378690.1], *Coniosporium apollinis* [XP_007784024.1], *Hortaea werneckii* [KAI6815255.1], *Fusarium oxysporum* [XP_018257415.1], *A. fumigatus* Z5 [KMK56800.1]; Zygomycota – *Phycomyces blakesleeanus* [XP_018291433.1], *Rhizopus microsporus* [PHZ11803.1]; animals – *Ostrea edulis* [XP_048766939.1], *Homo sapiens* [AAA35832.1], plants – *Arabidopsis thaliana* [NP_187716.1], *Zea mays* [CAA58147.1]. Amino acids identical in all are shaded black, those shared with *K. petricola* FER1 are shaded green. Blue – the four helices per protein (A-D), yellow – ferroxidase diiron center, red – ferrihydrite nucleation center; purple – iron ion channel.

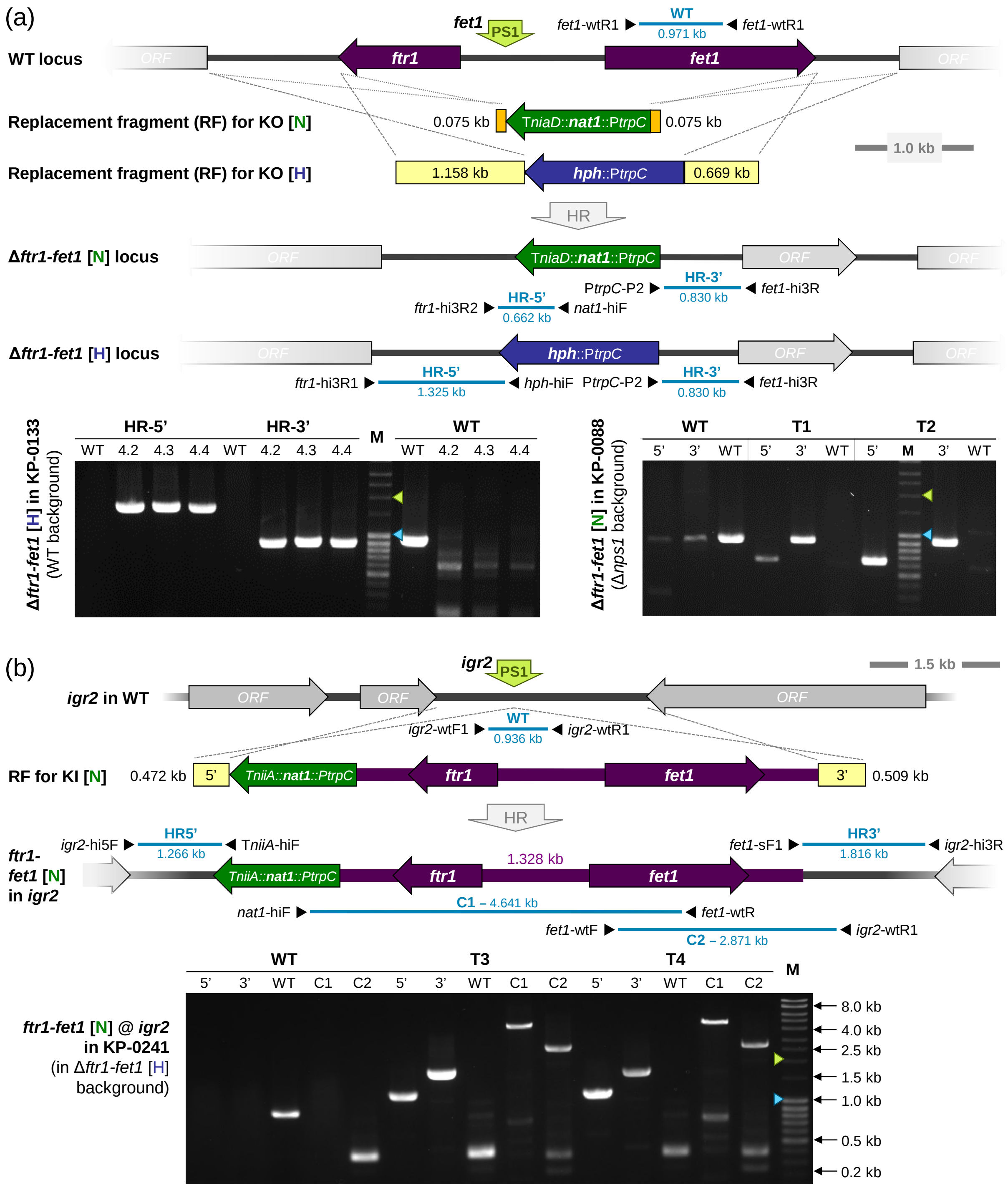

Figure S6: Deletion of *ftr1-fet1* and re-insertion of *ftr1-fet1* into Δ*ftr1-fet1* mutants.
(a) For generation of Δ*ftr1-fet1* mutants [KP-0133], protoplasts of the WT were transformed with the hygromycin resistance (hygR, H) cassette flanked by long-homologous (LH) sequences as donor DNA and the *in-vitro* assembled ribonucleoprotein RNP-*fet1* PS1 for a double strand break (DSB) in the shared promoter region (green arrow). For generation of Δ*nps1*/Δ*ftr1-fet1* double mutants [KP-0088], Δ*nps1* protoplasts were transformed with a nourseothricin resistance (natR, N) cassette flanked by short-homologous (SH) sequences and the *in-vitro* assembled RNP-*fet1* PS1. Homologous recombination (HR) leading to the replacement of the *ftr1-fet1* region was detected by diagnostic PCR. Primers and amplicon sizes are indicated. b) *Ftr1* and *fet1* regulated by the native bidirectional promoter were re-introduced in the intergenic region 2 (*igr2*) of Δ*ftr1-fet1* mutants yielding Δ*ftr1-fet1*::*ftr1-fet1* strains [KP-0241]. For this, an amplicon comprising *ftr1* and *fet1* with their 3'-noncoding regions (~0.6 kb) was assembled in pIGR2N to obtain pIGR2N-*ftr1-fet1* COM [pEC0081]. Protoplasts of the Δ*ftr1-fet1* mutant were transformed with the replacement fragment (RF) amplified from this plasmid and the Cas9- and sgRNA-delivering pAMA/ribo-*kpigr2* PS1 [pEC0059] for a DSB in *igr2*. Targeted integration (knock-in, KI) into *igr2* was detected by diagnostic PCR. M – MassRuler DNA Ladder Mix (green triangles: 2 kb, blue triangles: 1 kb). GenBank accession are: PP374642.1 (*ftr1-fet1*), OM802160.1 (*igr2*). See also Table S5, Table S6 and Table S7.

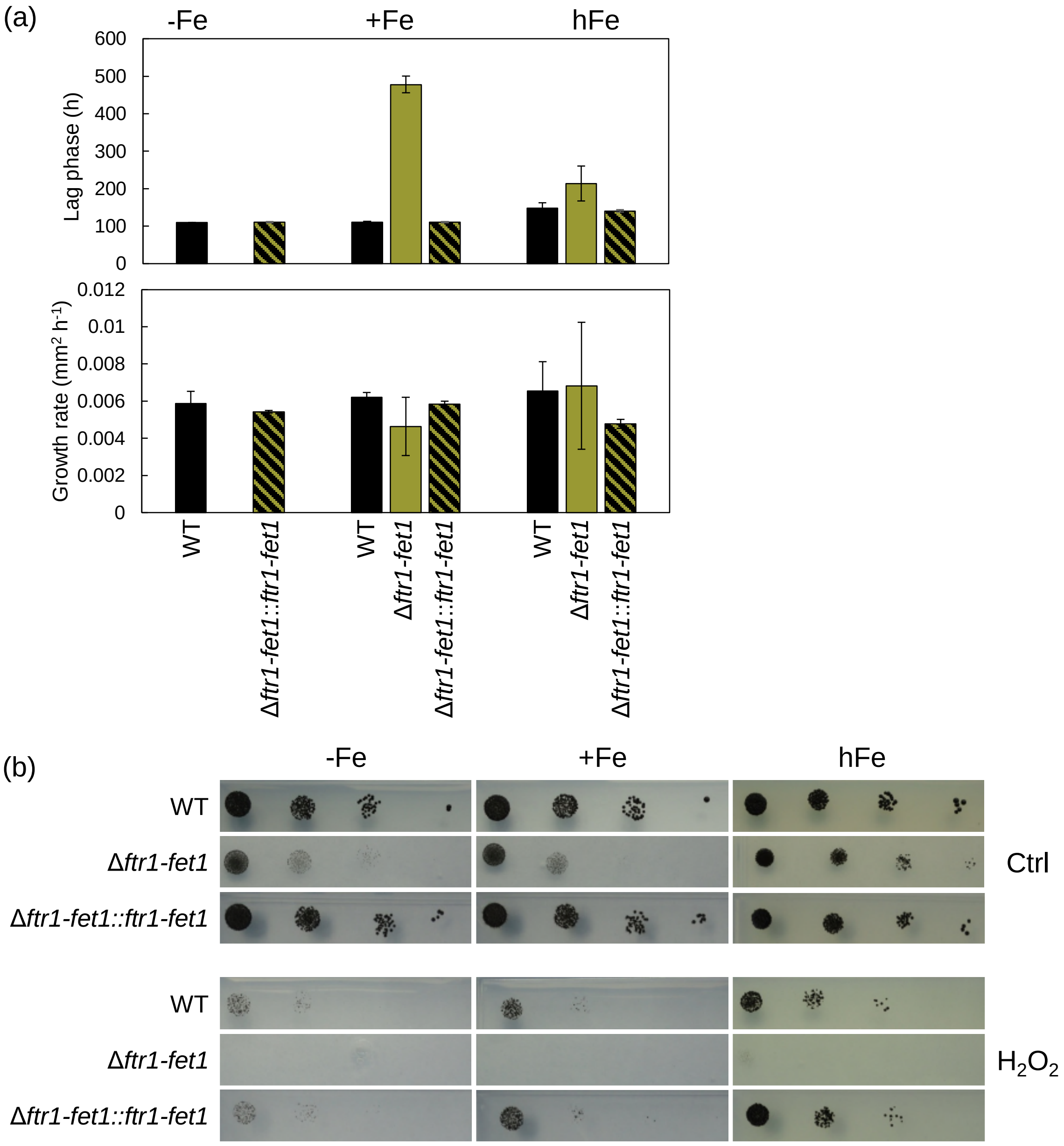

Figure S7: Growth under low-iron and oxidative stress conditions are restored in Δ*ftr1-fet1*::*ftr1-fet1*.
(a) Growth rates and lag phases were recorded by the ScanLag system when cultivated on solid medium with 0 µM iron (-Fe), 30 µM iron (+Fe) and 1,511 µM iron (hFe). Shown are the averages of three independent replicates with two times the standard error. The growth rate and lag phase could not be obtained for Δ*ftr1-fet1* on solid -Fe medium as growth was absent 30 dpi. (b) 10 µl of suspensions with 10^6, 10^5, 10^4, and 10^3 CFU ml^{-1} of the selected strains were point-inoculated on solid medium with different iron concentration without and with 2 mM (-Fe and +Fe) or 2.75 mM (hFe) H_2O_2. Images were taken 12 dpi.

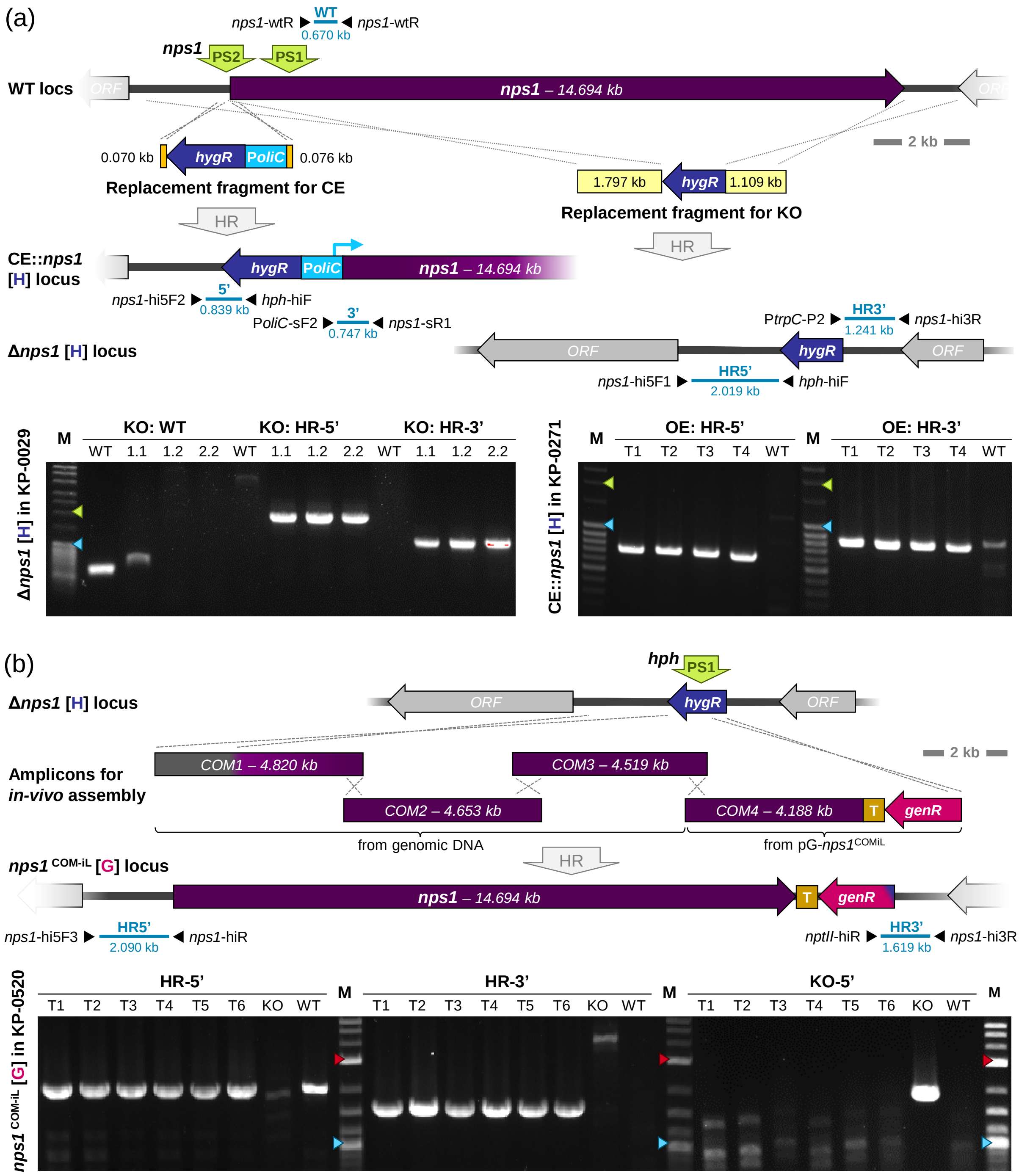

Figure S8: Deletion and constitutive expression of *nps1* and the re-insertion of Δ*nps1* in Δ*nps1*.
(a) For constitutive expression (CE) of *nps1* [CE::*nps1*, KP-0271], the strong P*oliC* from *A. nidulans* was inserted upstream of *nps1*. WT protoplasts were transformed with a replacement fragment (hygR cassette plus P*oliC* flanked by sequences homologous to the 5'-noncoding and the coding region of *nps1*) and the *in-vitro* assembled RNP-*nps1*$^{\text{PS2}}$ for a DSB upstream of the start. By HR, 0.150 kb of the 5'-noncoding region were replaced by P*oliC*. For generation of Δ*nps1* mutants [KP-0029], the *nps1* was deleted by transformation of WT protoplasts with a hygR cassette flanked by LH sequences and RNP-*nps1*$^{\text{PS1}}$ for a DSB in *nps1*. HR events leading to the knock out (KO) of *nps1* and the knock in (KI) of P*oliC* were detected by PCR using the indicated primer pairs. M – MassRuler DNA Ladder Mix. (b) Δ*nps1*::*nps1* [KP-0520] strains were generated by *in vivo* assembly of *nps1* in the Δ*nps1* locus, as the cloning of a complementation construct failed because of the length of *nps1*. Protoplasts of the Δ*nps1* mutant were transformed with four amplicons with overlapping regions, amplified from genomic DNA or the cloned pG-*nps1*^{COMiL} [pEC0491; contains the 3'-coding region of *nps1* fused to *B. cinerea* T*gluc* (T) and the geneticin resistance (genR) cassette P*trpC*::*nptII*::T*niiA*], and the Cas9- and sgRNA-delivering pAMA/tRNA-*hph*$^{\text{PS1}}$ for a DSB in *hph*. HR was mediated by the 5'-noncoding region of *nps1* and P*trpC* present in both hygR and genR cassettes, and resulted in the replacement of the hygR cassette with the P*nps1*::*nps1*::T*gluc*-genR construct, as confirmed by diagnostic PCR. M – 1 kb Plus DNA Ladder (red and blue triangles indicate the 3-kb- and 1-kb-large fragments, respectively). GenBank accession of *nps1* locus: PP374629.1. See also Table S5, Table S6 and Table S7.

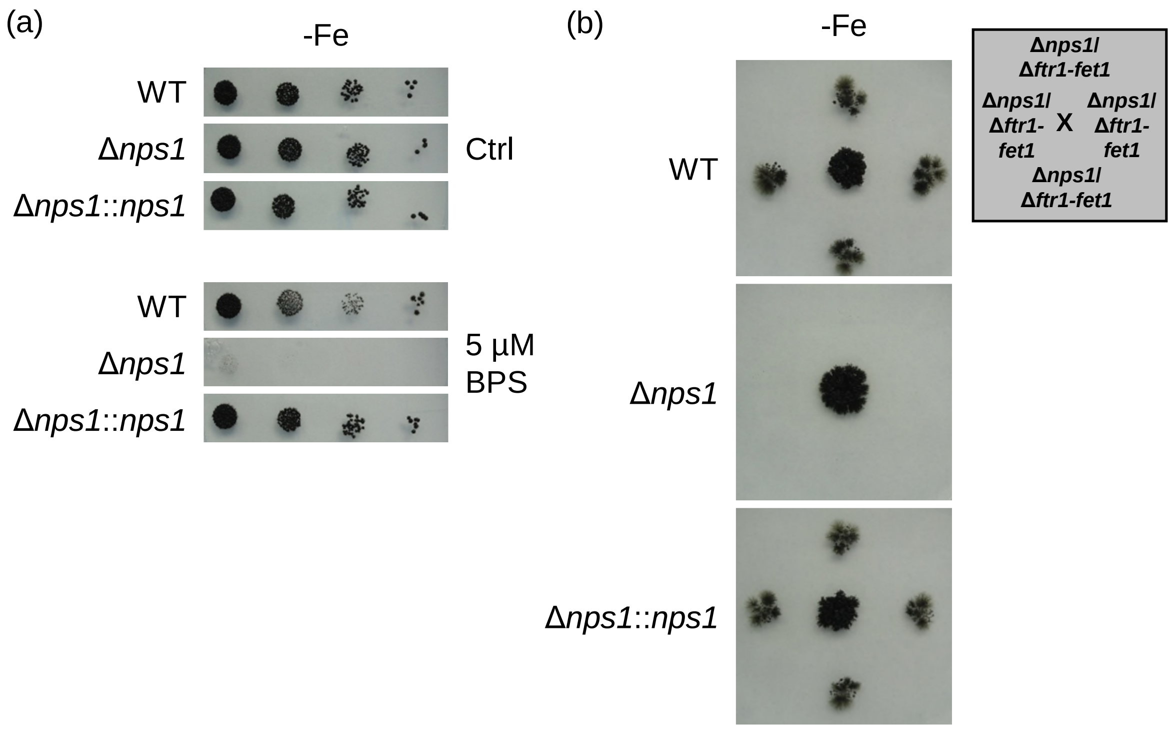

Figure S9: Growth under iron-depleted conditions and siderophore secretion are restored in Δ*nps1*::*nps1*. (a) 10-µl drops containing 10^4, 10^3, 10^2, and 10^1 CFU of the selected strains were point-inoculated on solid medium with 0 µM iron (-Fe), with and without 5 µM BPS. Images were taken 12 dpi. (b) 10-µl drops of 10^5 CFU ml^{-1} of the double mutant Δ*nps1*/Δ*ftr1-fet1* were point-inoculated next to 10-µl drops of either Δ*nps1*, WT or Δ*nps1*::*nps1* on iron-lacking medium. Growth of the double mutant Δ*nps1*/Δ*ftr1-fet1* indicates siderophore production by the neighboring 34 dpi. Both tests indicate that the complementation of the Δ*nps1* mutant was successful.

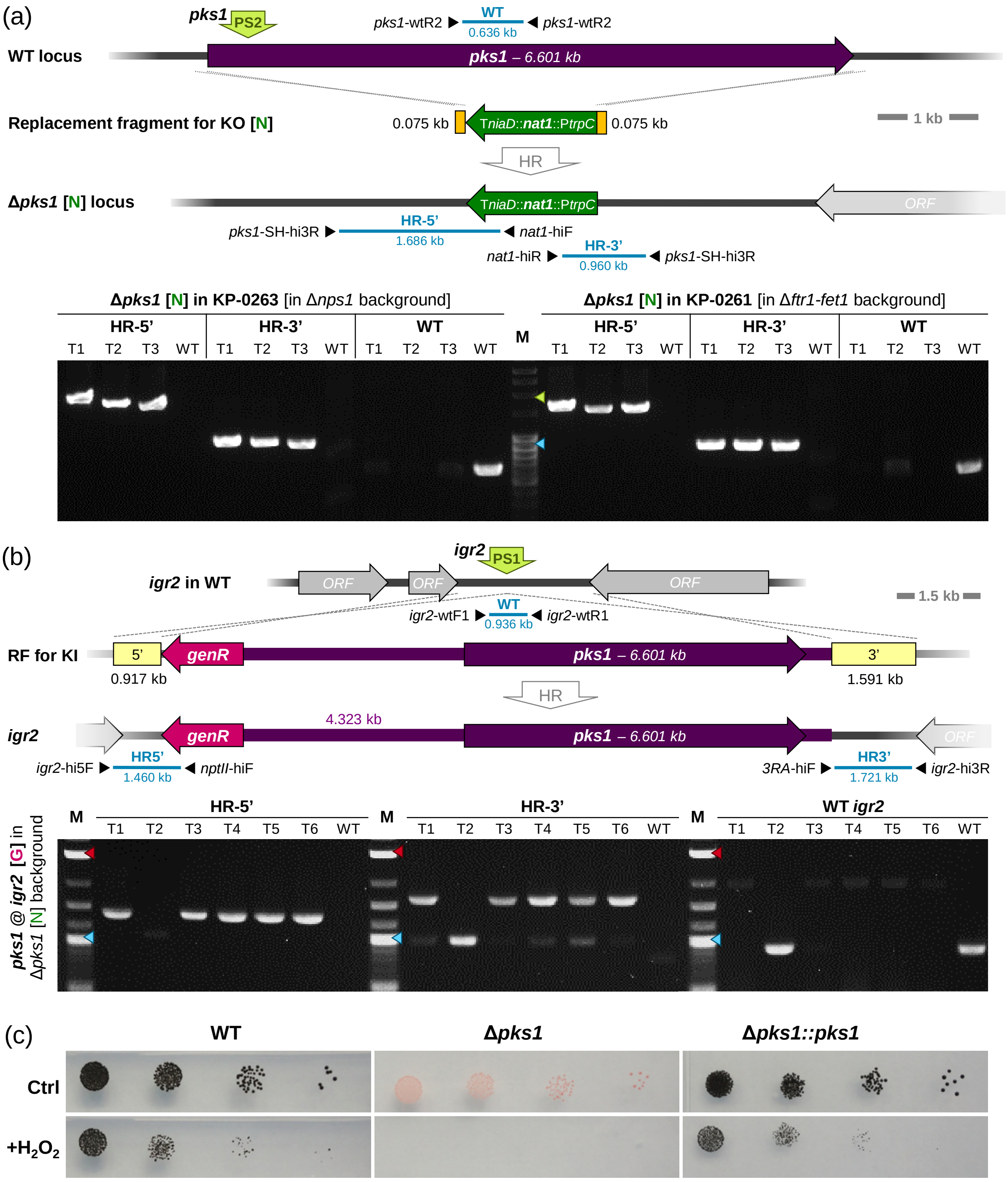

Figure S10: Deletion of *pks1* and complementation of the Δ*pks1* mutant.
(a) Generation of Δ*ftr1-fet1*/Δ*pks1* [KP-0261] and Δ*nps1*/Δ*pks1* [KP-0263] mutants by replacing *pks1* with a natR cassette in mutant backgrounds. For this, Δ*ftr1-fet1* and Δ*nps1* protoplasts were transformed with the natR cassette flanked by 75-bp-long sequences homologous to the noncoding regions of *pks1* and the Cas9- and sgRNA-delivering pAMA/ribo-*pks1* ^{PS2}. The replacement of *pks1* by the natR cassette was confirmed by diagnostic PCR in non-melanized transformants. M – MassRuler DNA Ladder Mix. (b) Generation of Δ*pks1*::*pks1* [KP-0507] strains by re-inserting *pks1* including 4.323 kb and 0.484 kb of its 5'- and 3'-noncoding regions in *igr2* of the natR Δ*pks1* mutant [KP-0033]. For this, two amplicons obtained from genomic DNA were assembled in pIGR2G yielding pIGR2G-*pks1*^{COM} [pEC0477]. Δ*pks1* protoplasts were transformed with the replacement fragment, which was isolated by digestion from pIGR2G-*pks1*^{COM}, and the Cas9- and sgRNA-delivering pAMA/ribo-*kpigr2* ^{PS1}. The expression construct in *igr2* was detected by diagnostic PCR. M – 1 kb Plus DNA Ladder. GenBank accessions: MT859418.1 (*pks1* locus), OM802160.1 (*igr2* locus). See also Table S5, Table S6 and Table S7. (c) Pigmentation and oxidative stress tolerance are recovered in Δ*pks1*::*pks1*. 10 µl of 10^4, 10^3, 10^2, and 10^1 CFU were spotted onto solid -Fe medium without or without 2 mM H_2O_2. Images were taken 12 dpi.

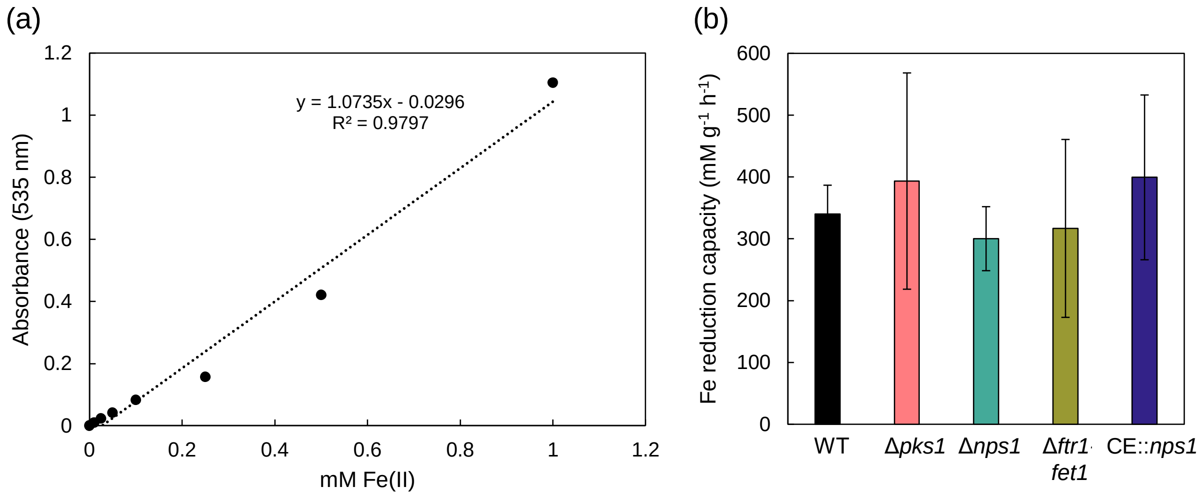

Figure S11: The standard curve of the iron reduction assay and iron reduction capacities of supernatants. (a) Standard curve of the reduction assay: Absorbance (535 nm) = 1.0735 $C_{Fe(II)}$ (mM) – 0.029. (b) Fe reduction capacity of the supernatants of selected strains. Absorbances were compared to the medium control, normalized by the dry weight of the biomass of the culture, the duration of the reaction (i.e. 3 h) and the volume of the culture. Note that these values are similar for all strains and generally ten times higher than the Fe reduction capacity of the biomass of these cultures (Figure 5).

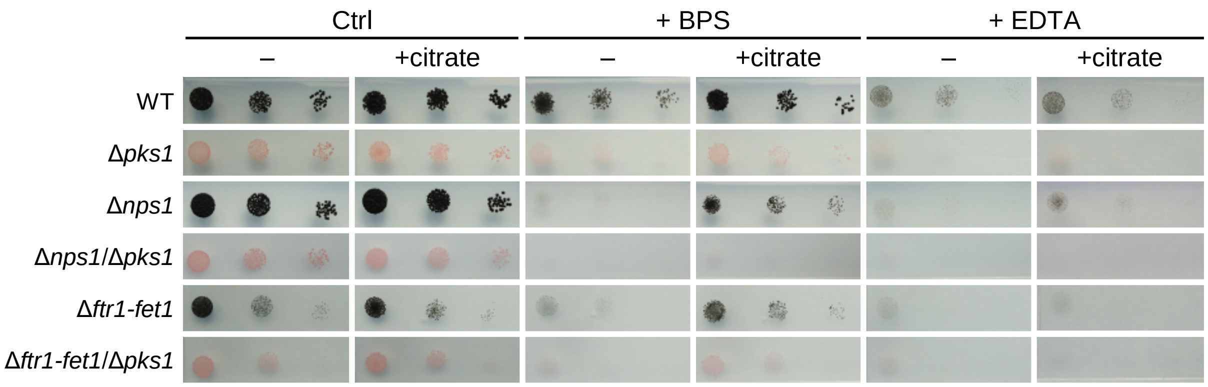

Figure S12: Former panel shown as Figure 5e.
This panel originates from the same experimental run as the data presented in Figure 3c and is provided here for transparency.

Supplementary References

1. Nai C, Wong HY, Pannenbecker A, Broughton WJ, Benoit I, de Vries RP, Gueidan C, Gorbushina AA. 2013. Nutritional physiology of a rock-inhabiting, model microcolonial fungus from an ancestral lineage of the Chaetothyriales (Ascomycetes). Fungal Genet Biol 56:54-66. https://doi.org/10.1016/j.fgb.2013.04.001

2. Voigt O, Knabe N, Nitsche S, Erdmann EA, Schumacher J, Gorbushina AA. 2020. An advanced genetic toolkit for exploring the biology of the rock-inhabiting black fungus *Knufia petricola*. Sci Rep 10:22021. https://doi.org/10.1038/s41598-020-79120-5

3. Grundlinger M, Gsaller F, Schrettl M, Lindner H, Haas H. 2013. *Aspergillus fumigatus* SidJ mediates intracellular siderophore hydrolysis. Appl Environ Microbiol 79:7534-7536. https://doi.org/10.1128/AEM.01285-13

4. De Lucca AJ, Boue S, Sien T, Cleveland TE, Walsh TJ. 2011. Silver enhances the in vitro antifungal activity of the saponin, CAY-1. Mycoses 54:e1-9. https://doi.org/10.1111/j.1439-0507.2009.01811.x

5. Lai YW, Campbell LT, Wilkins MR, Pang CN, Chen S, Carter DA. 2016. Synergy and antagonism between iron chelators and antifungal drugs in *Cryptococcus*. Int J Antimicrob Agents 48:388-394. https://doi.org/10.1016/j.ijantimicag.2016.06.012

6. Kubo I, Lee SH, Ha TJ. 2005. Effect of EDTA alone and in combination with polygodial on the growth of *Saccharomyces cerevisiae*. J Agric Food Chem 53:1818-1822. https://doi.org/10.1021/jf049363z

7. Yang D, Shi H, Zhang K, Liu X, Ma L. 2023. The antifungal potential of the chelating agent EDTA against postharvest plant pathogen *Botrytis cinerea*. Int J Food Microbiol 388:110089. https://doi.org/10.1016/j.ijfoodmicro.2023.110089

8. Liu F, Hansra S, Crockford G, Koster W, Allan BJ, Blondeau JM, Lainesse C, White AP. 2018. Tetrasodium EDTA is effective at eradicating biofilms formed by clinically relevant microorganisms from patients' central venous catheters. mSphere 3:e00525-18. https://doi.org/10.1128/mSphere.00525-18

9. Pinsky M, Kornitzer D. 2024. Genetic analysis of *Candida albicans* filamentation by the iron chelator BPS reveals a role for a conserved kinase-WD40 protein pair. J Fungi (Basel) 10:83. https://doi.org/10.3390/jof10010083

10. Albarouki E, Schafferer L, Ye F, von Wiren N, Haas H, Deising HB. 2014. Biotrophy-specific downregulation of siderophore biosynthesis in *Colletotrichum graminicola* is required for modulation of immune responses of maize. Mol Microbiol 92:338-355. https://doi.org/10.1111/mmi.12561

11. Robertson EJ, Wolf JM, Casadevall A. 2012. EDTA inhibits biofilm formation, extracellular vesicular secretion, and shedding of the capsular polysaccharide glucuronoxylomannan by *Cryptococcus neoformans*. Appl Environ Microbiol 78:7977-7984. https://doi.org/10.1128/AEM.01953-12

12. Kim SW, Park JK, Lee CH, Hahn BS, Koo JC. 2016. Comparison of the antimicrobial properties of chitosan oligosaccharides (COS) and EDTA against *Fusarium fujikuroi* causing rice Bakanae disease. Curr Microbiol 72:496-502. https://doi.org/10.1007/s00284-015-0973-9

13. Brilhante RSN, Costa ADC, Pereira VS, Fernandes MR, de Oliveira JS, Rodrigues AM, Camargo ZP, Pereira-Neto WA, Sidrim JJC, Rocha MFG. 2020. Antifungal activity of deferiprone and EDTA against *Sporothrix* spp.: Effect on planktonic growth and biofilm formation. Med Mycol 59:537-544. https://doi.org/10.1093/mmy/myaa073

14. Wiegand I, Hilpert K, Hancock RE. 2008. Agar and broth dilution methods to determine the minimal inhibitory concentration (MIC) of antimicrobial substances. Nat Protoc 3:163-175. https://doi.org/10.1038/nprot.2007.521

15. Christianson TW, Sikorski RS, Dante M, Shero JH, Hieter P. 1992. Multifunctional yeast high-copy-number shuttle vectors. Gene 110:119-22. https://doi.org/10.1016/0378-1119(92)90454-w

16. Staben C, Jensen B, Singer M, Pollock J, Schechtman M, Kinsey J, Selker E. 1989. Use of a bacterial hygromycin B resistance gene as a dominant selectable marker in *Neurospora crassa* transformation. Fungal Genet Rep 36:79. https://doi.org/10.4148/1941-4765.1519

17. Erdmann EA, Nitsche S, Gorbushina AA, Schumacher J. 2022. Genetic engineering of the rock inhabitant *Knufia petricola* provides insight into the biology of extremotolerant black fungi. Front Fungal Biol 3:862429. https://doi.org/10.3389/ffunb.2022.862429

18. Catanzaro I, Gerrits R, Feldmann I, Gorbushina AA, Onofri S, Schumacher J. 2024. Deletion of the polyketide synthase-encoding gene *pks1* prevents melanization in the extremophilic fungus *Cryomyces antarcticus*. IUBMB Life 76:1072-1090. https://doi.org/10.1002/iub.2895

19. Schumacher J. 2012. Tools for *Botrytis cinerea*: New expression vectors make the gray mold fungus more accessible to cell biology approaches. Fungal Genet Biol 49:483-497. https://doi.org/10.1016/j.fgb.2012.03.005

20. Von Blanckenburg F, Wittmann H, Schuessler JA. 2016. HELGES: Helmholtz Laboratory for the Geochemistry of the Earth Surface. Journal of large-scale research facilities JLSRF 2 https://doi.org/10.17815/jlsrf-2-141

21. Schuessler JA, Kämpf H, Koch U, Alawi M. 2016. Earthquake impact on iron isotope signatures recorded in mineral spring water. J Geophys Res-Sol Ea 121:8548-8568. https://doi.org/10.1002/2016jb013408

22. Omasits U, Ahrens CH, Muller S, Wollscheid B. 2014. Protter: interactive protein feature visualization and integration with experimental proteomic data. Bioinformatics 30:884-6. https://doi.org/10.1093/bioinformatics/btt607
